# Cell trajectory inference based on schrödinger problem and a mechanistic model of stochastic gene expression

**DOI:** 10.1038/s41540-026-00743-x

**Published:** 2026-05-21

**Authors:** Clémence Fournié, Elias Ventre, Ulysse Herbach, Aymeric Baradat, Olivier Gandrillon, Fabien Crauste

**Affiliations:** 1 Université Paris Cité, CNRS, MAP5, F-75006 Paris, France; 2 COMPO, Inria Méditerranée, Cancer Research Center of Marseille, Inserm UMR1068, CNRS UMR7258, UM105, Aix-Marseille Université, 13273 Marseille, France; 3 Université de Lorraine, CNRS, Inria, IECL, Nancy, France; 4 Univ Lyon, Université Lyon 1, CNRS UMR5208, Institut Camille Jordan, 43 Blvd du 11 Novembre 1918, , F-69622 Villeurbanne, France; 5 Univ Lyon, ENS de Lyon, Univ Claude Bernard, CNRS UMR 5239, INSERM U1210, Laboratory of Biology and Modelling of the Cell, 46 allée d’Italie Site Jacques Monod, F-69007 Lyon, France; 6 Inria, Lyon, France

**Keywords:** Biophysics, Computational biology and bioinformatics, Mathematics and computing, Physics, Systems biology

## Abstract

Cellular differentiation is the biological process that leads a cell to opt for a particular cellular identity. Recently, single-cell RNA sequencing has enabled the simultaneous measurement of gene expression levels at specific times for a large number of individual cells and a large number of genes. Repeating such measurements at different time points gives them access to the temporal variation, or transport, of a distribution on a gene expression space. The whole temporal trajectory of distributions thus characterizes the differentiation process at the population level, but trajectories of individual cells are still out of reach since most measurement techniques are destructive. The optimal transport theory that has been used so far to infer cellular differentiation trajectories from time-stamped single-cell RNA-seq data involves solving the so-called Schrödinger problem in its most common version. This implies assuming that cells move, in the gene expression space, by diffusion. Yet, real gene dynamics are much more complex. In the present work, we assume that mRNA dynamics are characterized by brief and important production of RNA, with long periods of inactivity in between, and consider the so-called Bursty model of gene dynamics. We use this model to define a reference process for the Schrödinger problem. By comparing the solutions of the Schrödinger problems with a Diffusive and a Bursty reference process, under different conditions, we show that the Bursty model provides a better approximation of the underlying gene dynamics than the standard Diffusive process when inferring cell trajectories.

## Introduction

While two cells in a multicellular organism may possess the same genome, they can express their genes differently, leading to distinct cellular outcomes. Cellular differentiation is the biological process that leads a cell to opt for a particular cellular identity^1^. In contemporary biology, the cellular identity is defined by the expression of cell markers, mostly surface proteins.

Recently, single-cell RNA sequencing (scRNA-seq) has enabled the simultaneous measurement of gene expression levels for up to millions of individual cells and tens of thousands of genes^2–4^. Thanks to this technique, a cell can be represented in a high-dimensional space, where each dimension corresponds to the expression level of a given gene, so the space dimension equals the number of genes. Repeating such measurements at different time points gives access to the temporal variation, or transport, of a distribution on a gene expression space. The entire temporal trajectory of the distribution characterizes the differentiation process at the population level, but trajectories of individual cells are still out of reach since the measurement process is destructive^5^. Hence, the goal to develop tools to infer trajectories between measurements to reconstruct the differentiation process has become crucial. Since cells are destroyed in the data acquisition process, inference of cell trajectories allows to determine potential cell fate under the assumption that the cell sample used at each measurement time is representative of the whole cell population.

In practice, gene expression is measured either as the number of mRNA molecules or the level of protein expression. Hence, the above-mentioned gene space is either an mRNA counts space or a protein level space. While the differentiation process is fundamentally a continuous process, most of the experimental measurements provide access only to discrete temporal distributions. In order to study a differentiation process and, more specifically, to infer differentiation pathways, many works focused on the reconstruction of the continuous transcriptomics evolution based on such distributions.

Schiebinger et al.^5^ were pioneers in using the optimal transport (OT) theory to infer such trajectories from time-stamped transcriptomics data, introduced in the Waddington Optimal Transport (WOT) algorithm. WOT enables the reconstruction of a “timeline” between two cell distributions measured at different times. WOT solves the OT problem in a standard way: by minimizing a cost function defined as the squared Euclidean distance between cells in a PCA-reduced space.

Since then, several works proposed methods for cell trajectory inference inspired by OT theory^6–17^. Authors of OT-based studies chose to work with quadratic OT, which consists in using the squared Euclidean distance as a cost in the OT problem (see Section Coupling between time points). More specifically, they used the so-called entropic approximation of OT—which is equivalent to solving the Schrödinger problem—in order to use the efficient Sinkhorn algorithm^18,19^. The Schrödinger problem consists of finding the stochastic process that is the closest to a given reference process while fitting the observations. Originally, the aim of the Schrödinger problem was to interpolate between cell distributions observed at discrete time points in order to reconstruct the full time-varying distribution. The mathematical theory supporting this problem ensures that this optimal interpolation is associated with an optimal coupling corresponding to the transition probability kernel between each pair of distributions of the underlying stochastic process. Thus, the destructive scRNA-seq measurements are perfectly aligned with the Schrödinger problem: the inferred coupling characterizes the optimal process describing the observed data under the hypothesis of the Schrödinger problem.

The reference process used in the Schrödinger problem is, in general, a Brownian motion, hence it is implicitly assumed that cell trajectories are close to diffusion. In some cases, distances different from the square Euclidean distance have been considered, mostly for taking into account additional information like lineage information^11,20^ or coexpression between genes^21^.

Some methods nevertheless used a different cost function when solving the OT problem. For instance, Liu et al.^17^ criticized the use of Euclidean distance when applying OT theory and proposed an implementation of a learned cost function in the OT problem based on the Sinkhorn algorithm^18^. However, they do not use OT theory to infer cell trajectories; instead, they focus on alignment of scRNA-seq datasets. Additionally, the learned cost is non-interpretable, a property that is shared by computational methods based on machine learning. In order to reduce the computational cost and gain interpretability, scEGOT^13^ combines OT with a cell state graph. By adding a Gaussian mixture distribution on each cell’s dots, scEGOT allows us to consider cell clusters instead of individual cells. The Gaussian mixture makes the results interpretable, however their objectives are clearly different from ours because the authors use Euclidean distance between Gaussian distributions to infer trajectories, relying once again on a standard approach.

The main idea of the present paper is to assert that in order to reconstruct cell differentiation trajectories, a more realistic, quantitative model of stochastic gene expression should be considered as a reference process. We focus on solving the Schrödinger problem while using an alternative reference process that would be closer to realistic gene dynamics. Indeed, the Diffusive process generates unrealistic dynamics for proteins and mRNA^22^. The Schrödinger problem is not seen here as an approximation of a transport problem, but as a way to identify a process close to an a priori quantitative model that would fit the observations.

When examined at the single-cell level, gene expression is undoubtedly a stochastic process^23–28^. The simplest model that can correctly reproduce experimental data distribution is the well-known two-state model of gene expression^29–31^, a refinement of the model introduced in 1991 by Ko^32^. In this model, a gene is described by its promoter, which can be either active or inactive—possibly representing a transcription complex being “bound” or “unbound” although it may be more complicated^33^—with mRNA being transcribed only during the active periods.

Genes may mechanistically influence the activation rate of other genes, thus creating a gene regulatory network (GRN). We consider an extension of the two-state model for several genes, that takes into account the GRN structure. For certain parameter values, mRNA dynamics are characterized by brief and important production of RNA, with long periods of inactivity in between: this regime corresponds to the widely observed phenomenon called transcriptional bursting^29^. Herbach et al.^34^ introduced a mathematical framework for modeling GRN driven by transcriptional bursting, which we call the Bursty model here. This model has been shown to reproduce realistic gene dynamics at the single-cell level^35,36^ and will provide our reference process.

In this work, we compare the performances of a Diffusive process, associated with standard OT theory, and a reference process based on the Bursty model to solve the Schrödinger problem, when initial and final distributions are based on realistic, simulated data. For the sake of clarity, in the following, we refer to our approach as the Bursty model-based inference method. We first explore the case of a single gene, then of a specific two-gene GRN, the toggle switch, and finally, more complex three-gene GRNs. We focus on the influence of some indicators, namely the time difference between the measurements of the two distributions and the number of cells in the distributions. We show that using the Bursty model as a reference process provides a better solution to the Schrödinger problem for all tested networks. Additionally, we show that the Bursty model-based inference method performs better than a Diffusion-based inference approach on a dataset made of mRNA counts for 41 genes.

## Results

We consider two distributions of protein counts obtained from a fixed number of cells, simulated from the Bursty model (see Section Gene dynamics) and measured respectively at times *t*_1_ and *t*_2_ (*t*_2_ > *t*_1_). Noticeably, we focus only on protein counts since protein dynamics, contrary to mRNA dynamics, allow to preserve the markovian property in the Bursty model therefore providing a rigorous mathematical framework. We solve the associated Schrödinger problem for several reference processes, including the Bursty process and the standard Diffusive process. Solutions of the Schrödinger problem are compared using an entropy function. If the two processes are similarly able to correctly infer trajectories, the entropy equals or nears zero.

When the final distribution is measured a “long” time after the initial distribution, the Bursty model has reached a so-called stationary state, and both initial and final distributions can be considered independent. In this case, trajectory inference should depend less on the selected reference coupling. Consequently, we focus our study on distributions in transient states, meaning the time difference *t*_2_ − *t*_1_ is small enough, and *t*_1_ is close to the initialization of the simulations, as represented in Fig. 12. Details are provided in section “Methods”.

Noticeably, since we are working with data that we generated, so-called simulated datasets, it is possible and efficient to define a ground truth. More precisely, it is convenient to use an identity-based ground truth, defined such that cell *i* at *t*_1_ corresponds to cell *i* at *t*_2_. However, when using experimental single-cell datasets, no ground truth is available, and only marginal observations can be used to infer the cell differentiation process. In this case, no reference exists to validate the result of any method.

### Schrödinger problem for a single gene

We first considered the case of a single gene with no feedback, as a toy model to illustrate the influence of the underlying model of gene dynamics on trajectory inference.

**Fig. 1:**
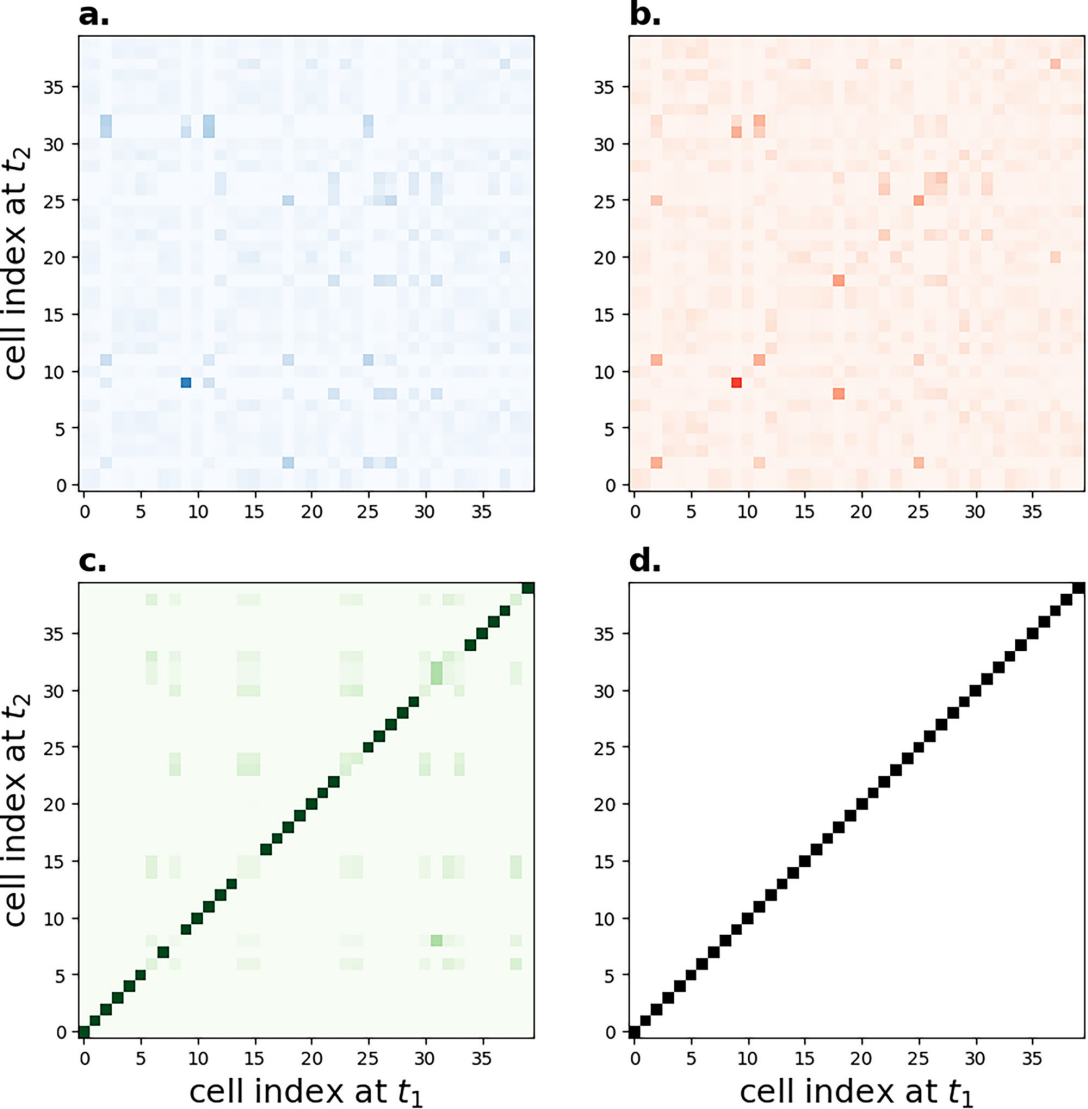
Solution of the Schrödinger problem for a single gene. **a** *D*_sch_. **b** *B*_sch_. **c** *RB*_sch_. **d** Ground truth. Each colored square represents the probability of the cell index at *t*_2_ starting from the cell index at *t*_1_ for *N* = 40 cells: the darker the box, the greater the probability. All matrices sum to 1. Parameters are given in Table 1.

Figure 1 shows the solutions of the Schrödinger problem computed with different approximated processes: a Diffusive model, *D*_sch_ (Fig. 1a), the non-reduced Bursty model, *B*_sch_ (Fig. 1b), and the reduced Bursty model, *RB*_sch_ (Fig. 1c). The ground truth of the simulated dataset (Fig. 1d) is a coupling of cells with the same index (cell *i* in the initial distribution is cell *i* in the final distribution). We observe that *RB*_sch_ yields the closest result to the ground truth of the simulated dataset (Fig. 1c), in the form of a very accurate transition matrix, while *D*_sch_ yields the furthest (Fig. 1a). A darker diagonal is observed for *B*_sch_ (Fig. 1b), meaning that this process better approximates the ground truth than *D*_sch_. Noticeably, a limitation of the representation in Fig. 1 is that the process could not be expressed (light color) and yet be relevant. For example, if two cells are close in protein space at both *t*_1_ and *t*_2_, then the process between those cells could be mixed up but relevant.

**Fig. 2:**
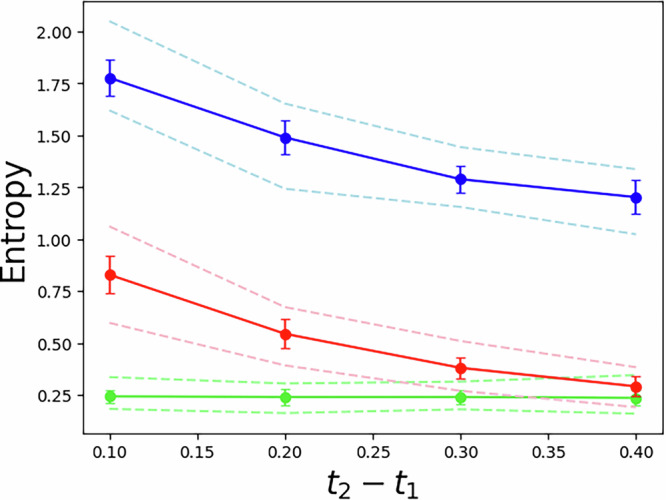
Entropies between reference couplings as a function of the time interval for a one-gene GRN. Mean and standard deviation (straight lines) of entropies *H*_*D*,*R*_ (blue), *H*_*B*,*R*_ (red) and *H*_*R*_ (green) are displayed for various values of the time difference *t*_2_ − *t*_1_, when the gene dynamics model is in a transient state. Additionally, minimum and maximum values over 50 iterations (dashed lines) are also displayed. Other parameters are detailed in Table 1.

Figure 2 presents the entropy between *D*_sch_ and *RB*_sch_, denoted by *H*_*D*,*R*_, and the entropy between *B*_sch_ and *RB*_sch_, denoted by *H*_*B*,*R*_ (see section “Performances of the models”). The curve *H*_*R*_ also appears in Fig. 2, it serves as a control by computing the entropy between the reference coupling and its approximation. The initial distribution is measured at time *t*_1_ = 0.1 h, and the final distribution is computed at various times *t*_2_ in the range [0.2; 0.5] h.

We observe that quantities *H*_*D*,*R*_ and *H*_*B*,*R*_ decrease as *t*_2_ − *t*_1_ increases and *H*_*R*_ remains constant. We also observe that *H*_*D*,*R*_ is always higher than the entropy between *B*_sch_ and *RB*_sch_, and remains much higher than *H*_*R*_ even for large values of *t*_2_ − *t*_1_ (here 0.4 h). Entropy *H*_*B*,*R*_ is always larger than *H*_*R*_, yet it is much smaller than *H*_*D*,*R*_, and for *t*_2_ − *t*_1_ = 0.4 h there is no difference between *H*_*B*,*R*_ and *H*_*R*_. Both the exact temporal distribution of the process and its approximation provide comparable results.

The study of this toy model highlights that using the exact temporal distribution of the reference process to solve the Schrödinger problem provides much better results than using the approximation of a gene dynamics model. However, it is a complex, most of the time impossible, task to find the analytical expression of this distribution for a GRN comprising at least two connected genes. Consequently, for a more relevant GRN, we will focus on the estimations provided by the reference process of the non-reduced Bursty model and the Diffusive process.

### Case study: the toggle switch

We focused on a two-gene GRN, called “toggle switch”, introduced in Section Example of GRN: the Toggle Switch and illustrated in Fig. 6b (blue GRN). This specific GRN consists of two self-activating genes that inhibit each other.

#### Influence of the time interval and the number of cells

We first investigated the influence of the time between measurements of the initial and the final distributions (i.e., *t*_2_ − *t*_1_) on the ability of two models, the Bursty and the Diffusive models, to correctly transport the cell distribution.

**Fig. 3:**
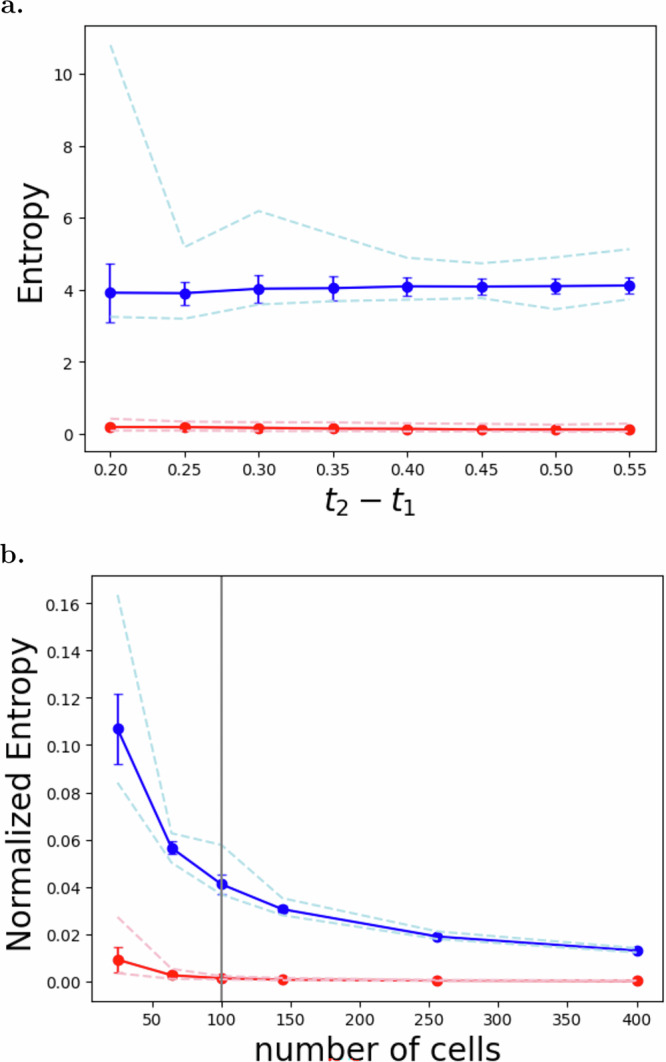
Entropies between reference processes for the toggle switch GRN, in the transient state. Mean and standard deviation (straight lines) of (**a**) *H*_*D*,*B*_ (blue) and *H*_*B*_ (red) as functions of the time interval *t*_2_ − *t*_1_, and (**b**) *H*_*D*,*B*_/*N* (blue) and *H*_*B*_/*N* (red) as functions of the number of cells *N*. Additionally, minimum and maximum values over 100 iterations are represented (dashed lines). The thick vertical gray line in Panel **b** is the default value *N* = 100 cells used in other figures. Other parameters are given in Table 1.

Figure 3a shows values of entropies *H*_*D*,*B*_ and *H*_*B*_ as functions of the time difference *t*_2_ − *t*_1_. The *H*_*D*,*B*_ curve is always above the *H*_*B*_ curve and remains approximately constant and independent of the time difference. The entropy between the two processes is the same for all *t*_2_ − *t*_1_ values: whatever the time difference, the Diffusive process infers trajectories with the same uncertainty. For the time difference *t*_2_ − *t*_1_ = 0.2 h, the entropy *H*_*D*,*B*_ has a larger standard deviation, due to one extreme value.

Although it is more relevant to focus on the transient state (Fig. 3a), it may be informative to question what happens in the stationary state. In this case, cells may either remain in their basin of attraction or switch to the other basin of attraction. The basin of attraction is a notion related to deterministic dynamics, consequently in the current framework where we consider stochastic models of gene dynamics it refers to the basin of attraction of the coarse approximation in the form of a system of ordinary differential equations of a piecewise-deterministic Markov process^37^. Results in the stationary state are illustrated in Supplementary File (Section 3), which highlight conclusions similar to the ones obtained for the transient state. Although the Diffusive model-based inference method is less accurate than the Bursty model-based inference method for inferring trajectories, its ability increases when the final distribution becomes independent of the initial one (later time measurements).

Figure 3b illustrates the influence of the number of cells *N* considered when measuring both initial and final distributions in the transient state. To that aim, the entropy has been normalized by the number of cells to avoid a bias due to the size of the collections in the computation of the entropy: the computation of the entropy (8) explicitly depends on the number of cells *N*. First, we observed that *H*_*B*_/*N* does not depend on the number of cells. Second, *H*_*D*,*B*_/*N* decreases as the number of cells increases. We observed (not shown) that the Diffusive process tends to couple a small sample of cells from the initial collection to all other cells in the final collection.

Both numerical investigations on the dependence of *H*_*D*,*B*_ and *H*_*B*_ on the length of the time interval *t*_2_ − *t*_1_ and the number of cells *N* highlighted a poor ability of the Diffusive model to reconstruct cell trajectories when the underlying model is the Bursty model of gene dynamics. These results are based on the use of the entropy to measure the differences between solutions of the Schrödinger problem. We then questioned the relevance of the entropy criterion and the parameter values of the Bursty model in reaching this conclusion.

#### Unspecificity of the entropy indicator and the distribution parameters

**Fig. 4:**
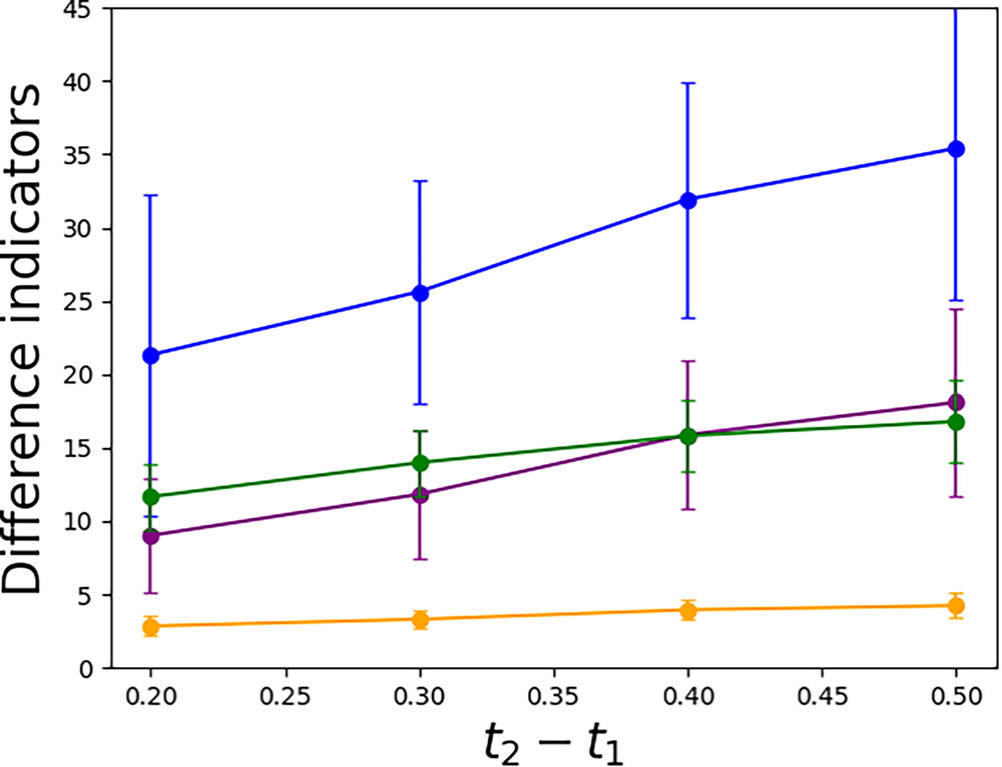
Difference indicators between reference processes as a function of the time interval for the toggle switch GRN. Mean and standard deviation of difference indicators comparing *D*_sch_ and *B*_sch_ normalized by the difference indicators comparing *B*_ref_ and *B*_sch_, respectively: Entropy (blue), total variation distance (yellow), Jensen–Shannon divergence (purple), and Frobenius norm (green). For standard deviations <0.1, error bars are not visible. Parameters are detailed in Table 1.

In Fig. 4, we compared the performance of different indicators when evaluating the difference between the Diffusive and the Bursty process. Figure 4 shows the mean and standard deviation of normalized indicators, computed as (*Indic* − *ControlIndic*)/*ControlIndic*, where *Indic* is either *H*_*D*,*B*_, *D*_*TV*_(*D*_*sch*_, *B*_*sch*_), *JSD*(*D*_sch_, *B*_sch_) or the Frobenius norm ∥*D*_sch_ − *B*_sch_∥_*F*_ (see Section Performances of the models) and *ControlIndic* its associated control measure (respectively, *H*_*B*_, *D*_*TV*_(*B*_ref_, *B*_sch_), *JSD*(*B*_ref_, *B*_sch_) and ∥*B*_ref_ − *B*_sch_∥_*F*_). We observe that for all indicators, the curves are all strictly positive and increasing. So, despite quantitative differences, all indicators yielded the same qualitative conclusion: the Bursty process provides a better trajectory inference than the Diffusive process.

To challenge the performance of the *B*_ref_ process, we generated the two distributions with a given GRN and used a different GRN for the reference process (mostly the same structure, but different interaction parameter values), that is, a different Bursty model. The use of simulated data provides full knowledge of the structure of the GRN and of its parameter values, however when dealing with experimental data the GRN is not known and can be at best inferred from the data, without any way to validate per se either the structure of the GRN or the parameter values. Hence, using a GRN that does not correspond to the data used to generate the initial and final distributions challenges the method and allows to test the robustness of the approach. In practice, we computed the initial distribution *μ* and the final distribution *ν* using the default parameter values of the toggle switch GRN, but we solved the Schrödinger problem with a different *B*_ref_ process (different gene interaction matrix).

**Fig. 5:**
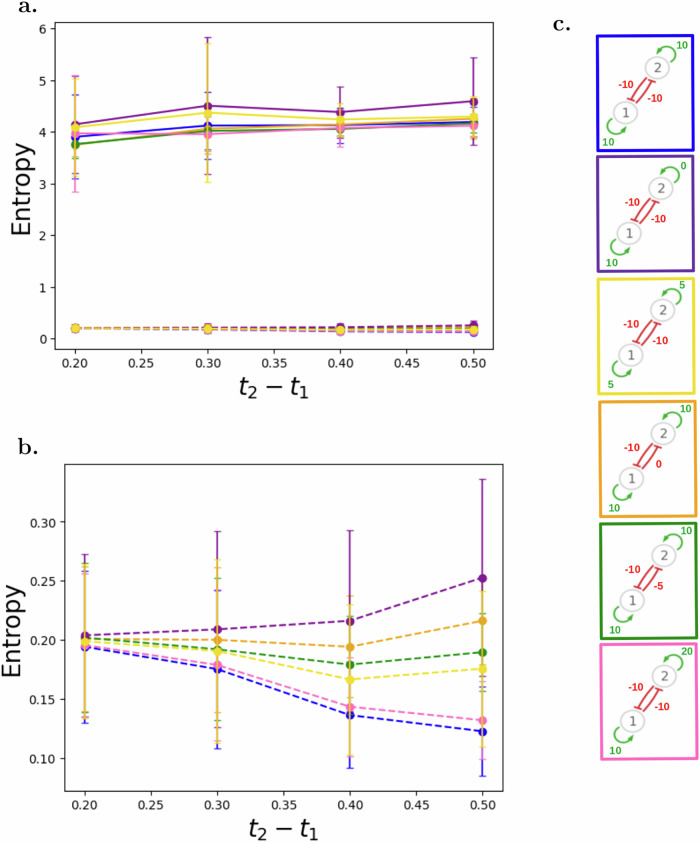
Entropy *H*_*D*,*B*_ as a function of the time difference *t*_2_ − *t*_1_ for different Bursty models, in the transient state. **a** Entropies *H*_*B*_ (dashed line) and *H*_*D*,*B*_ (straight line) means and standard deviations. Each color represents a set of interaction parameters of *B*_ref_ used to compute *H*_*D*,*B*_ (illustrated in panel **c**). **b** Zoom in on *H*_*B*_ values in panel **a** for *t*_2_ − *t*_1_ ∈ [0.2, 0.5]. **c** Two-gene GRN used to compute *B*_ref_. Each scheme depicts interactions between gene 1 and gene 2, with green arrows representing activation (*θ* > 0) and red arrows representing inhibition (*θ* < 0). The blue-squared GRN is the toggle switch used in previous figures. Parameter values are detailed in Table 1.

Figure 5a highlights that for all the two-gene-based Bursty models (listed in Fig. 5c), the curves are very close. The *H*_*D*,*B*_ curves are all higher than the *H*_*B*_ curves, indicating that any Bursty process, even one with parameter values different from the ones corresponding to the data, provides a better approximation than the Diffusive process. In Fig. 5b, one observes that even though *H*_*B*_ curves are nearly indistinguishable at early times, they become separated as *t*_2_ − *t*_1_ increases. We observed differences when parameter values of the GRN associated with self-activation were reduced (purple and yellow curves and GRN).

Overall, the Bursty model provides a framework for cell trajectory inference that performs better than standard diffusion.

We studied numerically the sensitivity of our method to hyperparameters (number of simulations *n*, standard deviation *ε* in (6), precision and truncation thresholds used to prevent zero values in the Sinkhorn algorithm) and to parameter values of the GRN, specifically focusing on degradation rates and basal activity levels (Supplementary File). No significant impact was observed on the entropy *H*_*D*,*B*_, pointing towards a weak sensitivity of the results on parameterization.

### Analysis for other GRNs

**Fig. 6:**
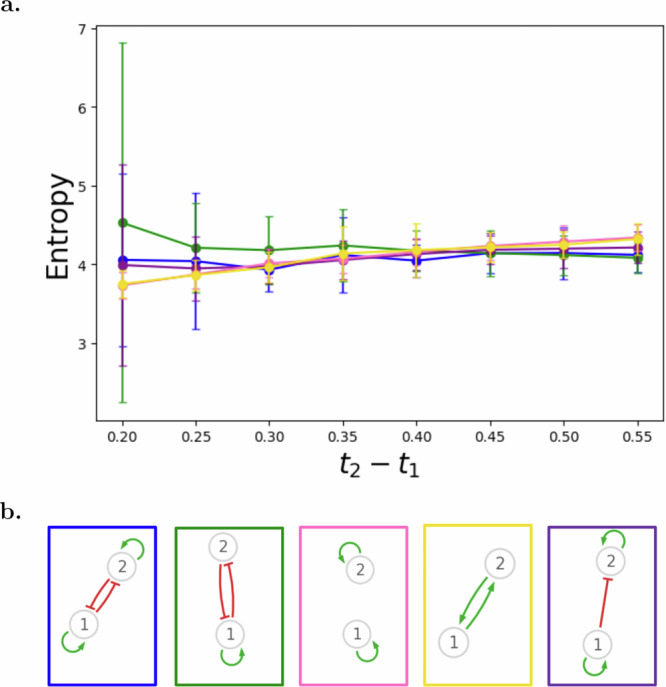
Entropy *H*_*D*,*B*_ as a function of the time difference *t*_2_ − *t*_1_ for various two-gene GRNs. **a** Entropy *H*_*D*,*B*_ in transient state, mean and standard deviation. Each color represents a GRN introduced in (**b**). Parameters are detailed in Table 1. **b** Schematic representations of two-gene GRNs used in (**a**). Green arrows represent activations (*θ* > 0) and red arrows represent inhibitions (*θ* < 0).

**Fig. 7:**
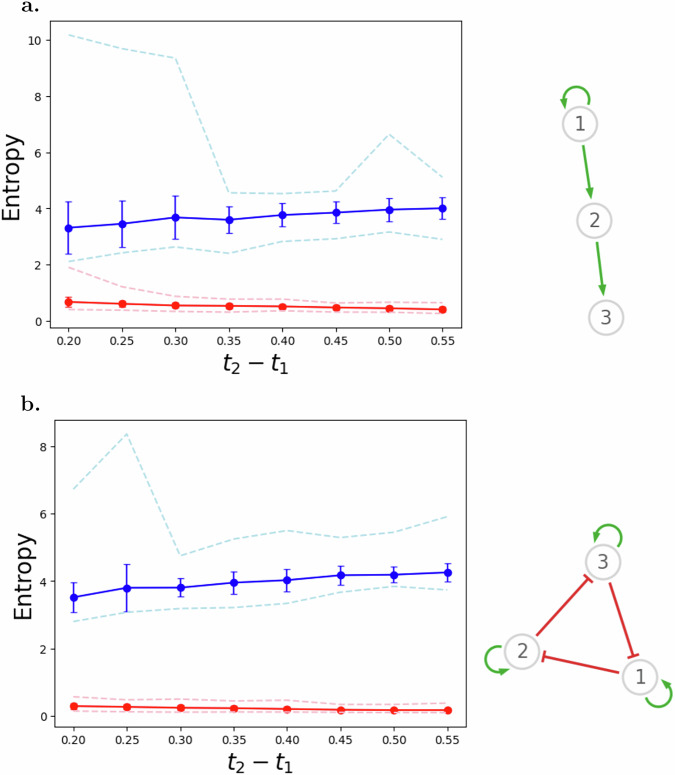
Entropy evolution as a function of the time difference *t*_2_ − *t*_1_ in the transient state for three-gene GRNs. **a** *H*_*D*,*B*_ mean and standard deviations (blue line with error bars) as well as min and max values (dash blue lines) and *H*_*B*_ mean and standard deviation (red line with error bars) and min and max values (dash red lines), and the associated three-gene GRN. The schematic representation of the GRN highlights positive interactions (activation) between genes. **b** *H*_*D*,*B*_ mean and standard deviations (blue line with error bars) as well as min and max values (dash blue lines) and *H*_*B*_ mean and standard deviation (red line with error bars) and min and max values (dash red lines), and the associated three-gene GRN (repressilator). The schematic representation of the GRN shows interactions between genes, with activations in green and inhibitions in red. Parameters are detailed in Table 1.

As illustrated in Fig. 11, the toggle switch model of gene dynamics exhibits what can be considered specific temporal dynamics. We subsequently challenged the Bursty process on other GRNs, first with various two-gene GRNs and then on three-gene GRNs. Results discussed hereafter and illustrated in Figs. 6 and 7 have been obtained using various GRNs to compute both the distributions (*μ* and *ν*) and the reference coupling. This contrasts with the results shown in Fig. 5, where only the reference coupling was modified by the GRNs, not the data.

Figure 6 shows the entropy *H*_*D*,*B*_ for different two-gene GRNs. For each GRN, the distributions and the Bursty process were computed from the corresponding Bursty model. The blue curve represents *H*_*D*,*B*_ values computed for the toggle switch. We observed that, whatever the two-gene GRN, the dynamics of *H*_*D*,*B*_ were similar, quantitatively and qualitatively: it was mostly constant as the time difference *t*_2_ − *t*_1_ increased, and the variability was higher for small time differences. Figure 7 shows the entropy *H*_*D*,*B*_ for two 3-gene GRNs. In the first one, gene 1 activates gene 2 which activates gene 3 (Fig. 7a); in the second one, the so-called repressilator model, gene 1 inhibits gene 2, gene 2 inhibits gene 3, and gene 3 inhibits gene 1 (Fig. 7b). Parameter values are the same than for the toggle switch (Table 1).

**Table 1:** Default parameter values

Description	Parameter	Value	Unit
Number of genes	*G*	2	genes
Number of cells	*N*	100	cells
Number of simulations	*n*	100	simulations
Initial conditions for mRNA counts	*IC*_*M*_	(0, 0)	mRNA
Initial conditions for protein counts	*IC*_*P*_	(0, 0)	protein
Smoothing standard deviation	*ε*	10^−4^	protein
Creation rate for proteins of gene *i*	*s*_*i*_	10	protein.h^−1^.mRNA^−1^
Minimum burst frequency of gene *i*	*k*_0,*i*_	0	h^−1^
Maximum burst frequency of gene *i*	*k*_1,*i*_	2	h^−1^
Basal activity of gene *i*	*β*_*i*_	5	h^−1^
Degradation rate of mRNA of gene *i*	*d*_0,*i*_	10	h^−1^
Degradation rate of protein of gene *i*	*d*_1,*i*_	5	h^−1^
Intensity of self-activation of gene *i*	*θ*_*ii*_	10	
Intensity of inhibition of gene *i*	*θ*_*ij*_	−10	
Initial time point in the transient state	*t*_1_	0.1	h
Final time point in the transient state	*t*_2_	0.5	h
Diffusion coefficient	*σ*^2^	0.001/2	protein^2^.h^−1^
Precision of the Sinkhorn algorithm		0.1	

Figure 7a shows a similar range of variations for *H*_*D*,*B*_ and *H*_*B*_ as observed for the two-gene GRNs. Variations about the mean were, however, more important than for the two-gene GRNs, as highlighted by error bars and minimum and maximum values (dashed lines). The *H*_*D*,*B*_ curve remains well above the *H*_*B*_ curve, similarly to what has been obtained for two-gene GRNs. The same results held for the Repressilator model (Fig. 7b), a standard three-gene GRN. Whatever the time difference, the mean of *H*_*D*,*B*_ was always higher than *H*_*B*_. For both GRNs, when *t*_2_ − *t*_1_ was small, the standard deviation of *H*_*D*,*B*_ was larger and the minimum and maximum values were widely separated. The two three-gene GRNs considered generated more differences between the Diffusive and the Bursty processes than the toggle switch model or any 2-gene GRN, as well as more variability.

In conclusion, for any two-gene or three-gene GRN, whatever the gene–gene interactions considered, the Bursty process always performed better than the standard Diffusive process in correctly transporting the distribution at time *t*_1_ into the distribution at time *t*_2_.

### Application to experimental data from ref. ^38^

While previous sections have been devoted to validating the Bursty-based inference method on small GRN and simulated data, here we apply the method to an experimental dataset. We use a time-stamped in vitro dataset from ref. ^38^ made of mRNA counts obtained from scRNA-seq on mouse embryonic stem cells. A GRN in the form of a bursty model has been inferred from these data by ref. ^36^, therefore, we use this GRN and the parameter values obtained during the inference procedure to compute our reference process *B*_ref_.

**Fig. 8:**
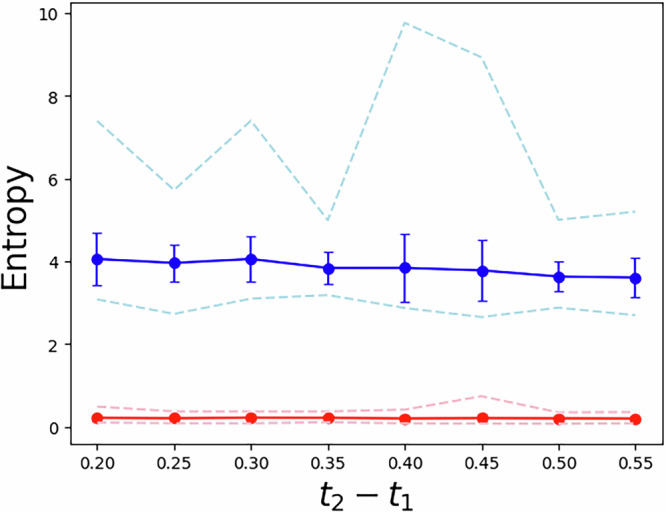
Entropies between reference processes for the toggle switch GRN for mRNA data. Mean and standard deviation (straight lines) of *H*_*D*,*B*_ (blue) and *H*_*B*_ (red) are displayed as functions of the time interval *t*_2_ − *t*_1_. Additionally, minimum and maximum values over 100 iterations are represented (dashed lines). Parameters are given in Table 1.

Since all previous investigations were performed on protein data, we first check whether or not the Bursty model-based inference method would also perform well on simulated mRNA data. Similarly to what we did in Fig. 3a, considering the toggle-switch GRN, we investigated the impact of time difference *t*_2_ − *t*_1_ on the entropy *H*_*D*,*B*_ but this time using only simulated mRNA data. Figure 8 shows that the *H*_*D*,*B*_ curve is higher than the *H*_*B*_ curve. Contrary to what is observed with protein level distributions, the *H*_*D*,*B*_ curve slightly decreases as *t*_2_ − *t*_1_ increases, yet remains far above the control curve. mRNAs are produced and degraded more rapidly than proteins, accelerating the approach to the stationary state. The same conclusions then hold, whether mRNA or protein data are used to infer cell trajectories.

We focus now on experimental mRNA data from ref. ^38^. When applying the Bursty model-based inference method to experimental data, no ground truth is available therefore, in a general context, standard or recently proposed methods—such as cluster-to-cluster fate probabilities^39^, ancestral/source compositions^5^—may allow a posteriori validation by focusing on checkable quantities obtained from the identified coupling, provided that the size of the sample is large enough. Here, in order to apply our Bursty model-based inference method, we chose to focus on its predictive power. We first computed an mRNA-based version of the process *B*_sch_ between time points *t*_1_ = 0 and *t*_3_ = 72 h, and estimated the distribution *ρ*_est,36_ at *t*_2_ = 36 h (see Section Application to ref. ^38^ dataset and Supplementary File, Section 4). Next, we computed the Earth Mover’s Distance (EMD) between the experimental distribution at 36h and, on one side, *ρ*_est,36_, denoted by EMD_*B*_, and on the other side, the distribution obtained using regularized OT, *ρ*_D,36_, denoted by EMD_*D*_.

**Fig. 9:**
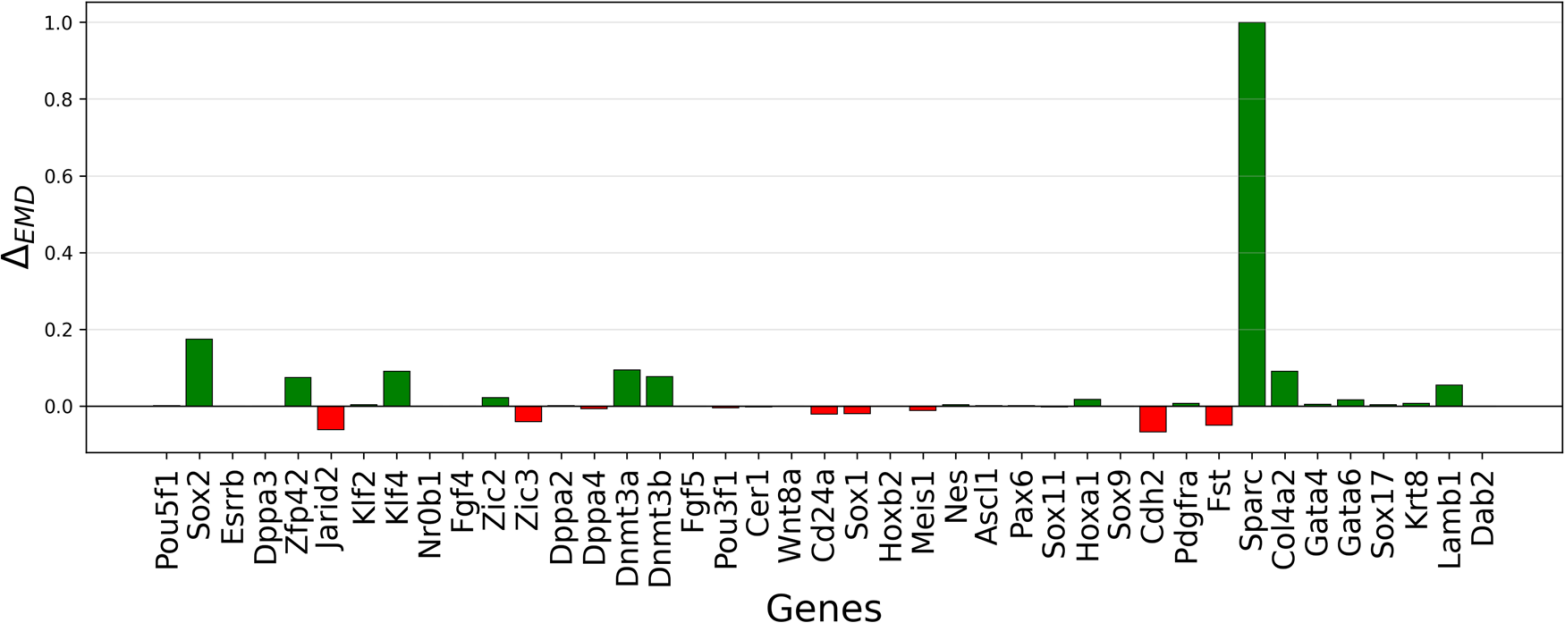
Gene-by-gene differences between EMD_*B*_ and EMD_*D*_ at *t*_2_ = 36 h, using data at *t*_1_ = 0 h and *t*_3_ = 72 h. Bar plots are green and positive when the Bursty model-based inference method performs better; they are red and negative when the Diffusion-based inference method performs better.

Figure 9 shows *Δ*_EMD_ = EMD_*D*_ − EMD_*B*_ for each of the 41 genes in the dataset. It allows a measure of the performance of the Bursty model-based inference method compared to the Diffusion-based inference method. Green bar plots correspond to a positive *Δ*_EMD_, i.e., EMD_*B*_ is smaller than EMD_*D*_, and red bar plots correspond to negative *Δ*_EMD_, i.e., when EMD_*B*_ is larger than EMD_*D*_. Heights of bar plots quantify the contributions of Bursty model-based versus Diffusion-based inference methods.

For 11 genes, *Δ*_EMD_ = 0. This means either that both *EMD*_*B*_ and *EMD*_*D*_ equal 0 (genes *Fgf5*, *Cer1*, *Ascl1*, and *Sox9*), or that they are equal (*Esrrb*, *Dppa3*, *Nr0b1*, *Fgf4*, *Wnt8a*, *Pax6*, and *Gata4*). In the first case, both methods are able to correctly predict the gene expression level, which is close to zero for the considered genes (no expression). In the second case, both methods similarly predict the gene expression level. Noticeably, gene distributions that do not evolve much between *t*_1_ = 0 h and *t*_3_ = 72 h display a small EMD.

Out of the remaining 30 genes, for 22 genes the Bursty model-based inference method performs better. Values of *EMD*_*B*_ and *EMD*_*D*_ for each gene are displayed in Supplementary File (Suppl. Fig. 4). Noticeably, the large bar-plot of gene *Sparc* indicates that the distance between the two *EMD* is negative and rather large but, in that case, both *EMD*_*B*_ and *EMD*_*D*_ are also large (both methods have difficulties correctly predicting this gene dynamics). For genes *Col4a2* and *Lamb1*, differences between EMD_*D*_ and EMD_*B*_ are also negative, yet smaller than for *Sparc*, but in that case, both *EMD* are small (both methods perform well in predicting these gene dynamics, the Bursty model-based inference method performing better).

One may note that we only focus here on an illustrative example where we predict the distribution at 36 h by using distributions at 0 and 72 h. However, the results are representative of other predictions that could be tested (not shown). Sometimes both Bursty model-based and Diffusion-based inference methods compare well (in particular in the early times, when only few genes drive the dynamics and their levels of expression do change slowly over time), nevertheless, the above results show that the Bursty model-based inference method can be applied on experimental data and performs better than the Diffusion-based method.

## Discussion

We explored the possibility that the Bursty model of gene dynamics would represent an improved alternative to the standard diffusion model used to infer trajectories in OT theory. Based on distributions of protein counts, the Bursty model proved indeed quite efficient in capturing cell trajectories more accurately than the Diffusive model (Fig. 1). This would be expected if we considered cells moving around in a Waddington’s landscape^40^ whose shape is constrained by the GRN structure^26,41^. In this case, diffusion could not easily represent the variables acceleration and deceleration due to valleys and hills. Noticeably, we assumed for the sake of simplicity the same number of cells in both initial and final distributions, yet our entire analysis could be extended to the case with different cell numbers by normalizing the distributions.

When considering a toy model made of only one gene, we concluded that using the exact time-dependent distribution of the process was the best alternative to diffusion. Although expected, this result indicates that it is possible to very efficiently infer cell trajectories in a transcriptomic space. However, the exact distribution is only explicit for one gene. Extending the reference coupling *RB*_ref_ to GRN with multiple genes seems a very challenging task, probably impossible to do for complex gene interactions. An approximation of this distribution could be considered, yet the approximation might rapidly become coarse.

For a one-gene GRN, the computation of *RB*_ref_ and *D*_ref_ couplings is almost instantaneous on a personal computer (64 GB RAM and 13th Gen Intel(R) Core(TM) i7-13700H), whereas the computation of *B*_ref_ takes approximately 7s (value averaged over 100 simulations). Increasing the number of genes does not impact the computational time for *D*_ref_, but it increases for *B*_ref_. The computational time is proportional to the number of genes in the GRN, with almost no time-variability from one simulation to another, for two-gene up to six-gene GRNs where genes self-activate and inhibit the other genes (similar to the toggle switch model). For two genes, the computational time is 12 s, for three genes, it is 13 s, for four genes, 14 s, for five genes 15.5 s, and for six genes 17 s per simulation of the *B*_ref_ coupling, on a computational server (high-throughput computing platform with 1 CPU and 2 GB memories). For most applications, such computational time would be acceptable. If needed, computations of *B*_ref_ coupling can be parallelized, for instance, when the number of simulations, the number of cells, or the number of genes is large. For the application to experimental data in Section Application to ref. ^38^ dataset, the computation of the *B*_ref_ coupling took 23 min using parallelization and 168 min without parallelization.

The full two-state model provides both mRNA and protein counts. Since experimentally available scRNA-seq datasets provide mRNA counts, it would have been appropriate to focus only on mRNA counts. However, it is not possible to reduce the Bursty model to an mRNA-only model and to preserve mathematical results, due to the fact that mRNA are typically much less stable than proteins and thereby would not preserve the Markovian property of the GRN. We can, however, reduce the Bursty model to a protein-only model that is well defined, along with quasi-steady-state distributions of mRNA counts conditionally on proteins^34,42^. Moreover, ref. ^43^ showed that for such a model, it is still possible to define and solve a Schrödinger problem. We therefore decided to focus on distributions of protein counts when benchmarking on simulated datasets.

In section Application to experimental data from ref. ^38^”, we applied our Busty model-based inference method on experimental data consisting of mRNA counts. We showed first that despite the above arguments in favor of preferentially using protein data, the Bursty model can also be applied to simulated mRNA data and provides in this case similar performances. To use our approach on an experimental dataset, it is first required to get a GRN, associated with the data, that is used to compute the reference coupling. This motivated the use of the dataset from ref. ^38^, since ref. ^36^ had already inferred a GRN from this dataset using CARDAMOM. The inference and calibration of the mechanistic model with CARDAMOM are based on a two-step procedure, with bursting parameters being identified in the first step^36,41^. Noticeably, other algorithms have been proposed to infer the bursting kinetics from single-cell RNA sequencing data^44–47^, the more recent ones benefiting from AI-based methods to infer kinetics and the method proposed in ref. ^47^, relying on the use of the Telegraph model, as does our method. Importantly, even if the underlying GRN structure is not perfectly known, which is expected from using inference methods to obtain the GRN, we established in Section Analysis for other GRNs (see, for instance, Fig. 5 and Supplementary File) that the Bursty model-based inference method is not very sensitive to parameter values of the GRN and correctly catches the trajectories even though the GRN used to compute the reference coupling is not correct.

When trying to predict an intermediate observation with ref. ^38^, the interpolation made with the Bursty model-based inference method performed better than the one made with the diffusion-based inference method. It is, however, worth noticing that the Diffusion-based inference method works well when observations are made on a short time period and when gene activity is limited. When observations are made on longer periods of time and genes exhibit important activity, the Bursty model-based inference method performs systematically better. Altogether, the results presented in this article thus pave the way to the extension of GRN inference algorithms to methods combining GRN and trajectory inference in an integrated framework.

## Methods

### Gene dynamics

We model gene expression using the framework introduced by ref. ^34^, and later simplified in the bursty regime^41,42,48^. In this model, a gene is characterized by its amount of mRNA and its level of proteins: mRNA is synthesized through stochastic transcriptional bursts, and degraded at rate *d*_0_; proteins are synthesized from mRNA at rate *s*_1_ and degraded at rate *d*_1_. The Bursty model is a piecewise-deterministic Markov process, with deterministic part given by

1 $$\left\{\begin{array}{l}\mathop{M}\limits^{.}(t)=-{d}_{0}M(t),\\ \mathop{P.}\limits^{.}(t)={s}_{1}M(t)-{d}_{1}P(t),\end{array}\right.$$

where *M*(*t*) and *P*(*t*) are continuous approximations of the quantity of mRNA molecules and the amount of protein, respectively, at time *t*, and a stochastic part consisting of mRNA bursts occuring at random times with rate *k*_on_ and random sizes that follow an exponential distribution^41,48^.

**Fig. 10:**
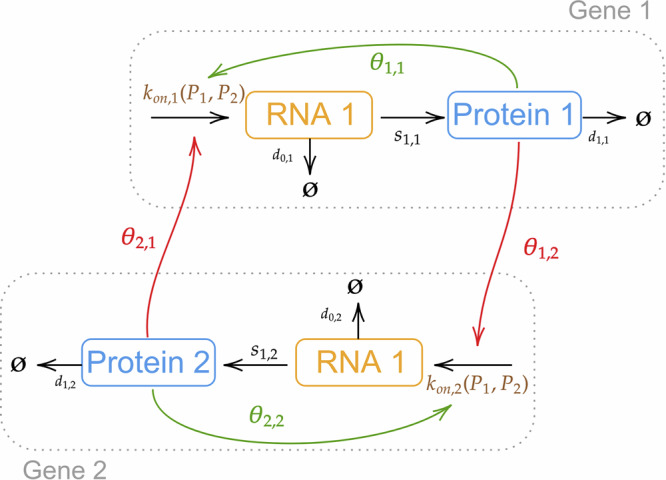
Schematic representation of a two-gene GRN based on the Bursty model of gene dynamics. For each gene *i* = 1, 2, mRNA synthesis is activated with a rate *k*_*on*,*i*_(*P*_1_, *P*_2_), defined in (2), and mRNA and protein dynamics follow system (1). Parameters of the GRN are described in the text. In the case of the toggle switch model, the GRN consists of 2 genes that self-activate (*θ*_1,1_ > 0 and *θ*_2,2_ > 0, green arrows) and inhibit each other (*θ*_2,1_ < 0 and *θ*_1,2_ < 0, red arrows).

Within a cell, genes interact with each other via a GRN. Hence, a gene can be activated or inhibited by itself but also by other genes in the GRN. A burst is assumed to occur for gene *i* at a rate *k*_on,*i*_(*P*_1_, …, *P*_*G*_), where *G* is the number of genes in the GRN and (*P*_1_, …, *P*_*G*_) is the vector of protein levels of all genes in the GRN. The burst rate of gene *i* accounts for a basal activity parameter *β*_*i*_ and the influence of other genes in the GRN, expressed via a gene–gene interaction matrix $\theta ={\{{\theta }_{ji}\}}_{j,i\in \{1,\ldots ,G\}}$^48,49^, so that

2 $${k}_{on,i}({P}_{1},\ldots ,{P}_{G})=\frac{{k}_{0,i}+{k}_{1,i}\exp ({\beta }_{i}+{\sum }_{j=1}^{G}{\theta }_{ji}{P}_{j})}{1+\exp ({\beta }_{i}+{\sum }_{j=1}^{G}{\theta }_{ji}{P}_{j})},$$

where *k*_0,*i*_ and *k*_1,*i*_ correspond respectively to the minimum and maximum burst frequencies of gene *i*, and *θ*_*ji*_ is the intensity of gene *j* action on gene *i*^34^. Parameters *θ*_*ji*_ can be positive or negative, depending on whether gene *j* activates or inhibits gene *i* (Fig. 10).

#### Example of GRN: the toggle switch

We consider a standard GRN made of two genes, the so-called “toggle switch” model. The two genes self-activate and inhibit each other, i.e.,

$${\theta }_{11}={\theta }_{22}\ge 0\,\,\mathrm{and}\,\,{\theta }_{12}={\theta }_{21} < 0.$$

**Fig. 11:**
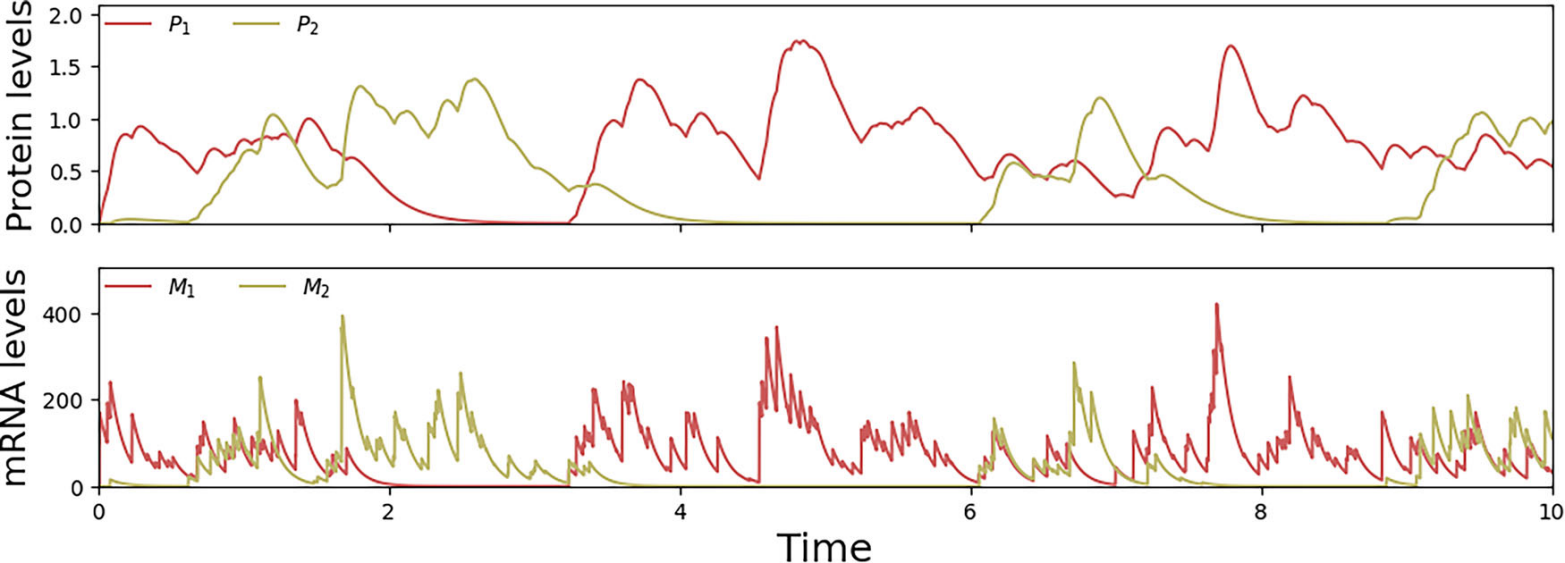
Temporal evolution of protein and mRNA levels for the toggle switch model. Dynamics of one gene are illustrated in yellow, the other in red. Protein levels are displayed on the upper panel, while mRNA dynamics are displayed in the lower panel.

This GRN is illustrated in Fig. 10, while Fig. 11 illustrates the temporal evolutions of mRNA and proteins for a toggle switch GRN where gene dynamics obey the Bursty model, computed with parameter values given in Table 1. A deterministic toggle switch model would favor one gene over the other, depending on initial conditions of the system. In this stochastic version of the toggle switch, one observes that one gene activity always prevails, yet the dominant gene switches stochastically.

More generally, by modifying the values of the interaction parameters *θ*_*ji*_ in model (1)-(2), any two-gene GRN can be implemented (see, for example, Fig. 6). Extensions to three-gene GRNs can be straightforwardly implemented (see, for example, Fig. 7).

#### Simulations of protein dynamics

**Fig. 12:**
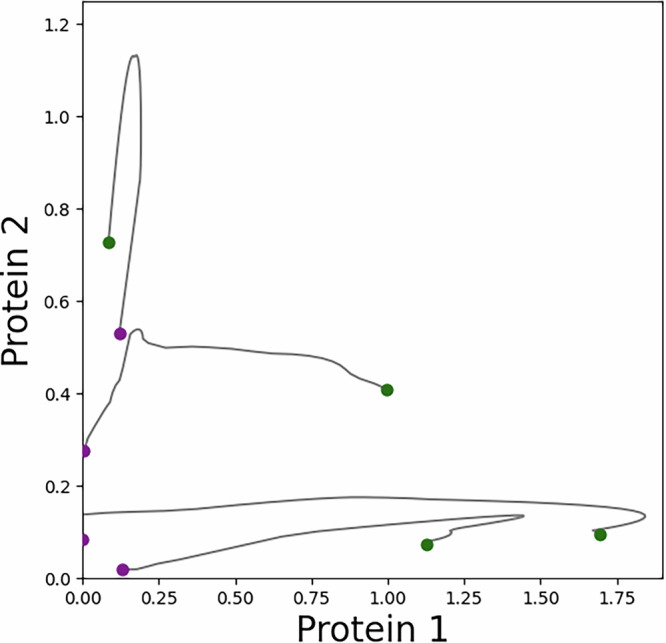
Cell trajectories of four cells driven by a toggle switch. Initial (time *t*_1_ = 0.1 h) location of cells is displayed as purple dots, while their final (time *t*_2_ = 0.5 h) location is represented by green dots. Each gray line is the temporal trajectory of each cell. Parameters are given in Table 1.

Computations of the temporal evolution of protein and mRNA counts were performed using the Python package Harissa^49^, which implements an exact simulation procedure for the Bursty model^42^ (Fig. 11). This allows to simulate one GRN, hence simulating *N* times the evolution of a GRN results in *N* independent cell trajectories (Fig. 12).

In order to mimic biological experiments aimed at measuring a GRN activity at given observation times, we simulate *N* times the GRN with initial conditions *IC*_*M*_ and *IC*_*P*_ (see Table 1), select a simulation time *t*, and extract all mRNA and protein counts for all genes.

Throughout this work, we focus on distributions of protein counts only, yet the case of mRNA distributions is discussed in the last section. We denote by *μ* the distribution of *G* protein counts in a population of *N* cells at time *t* = *t*_1_, and *ν* the distribution of *G* protein counts in the same population at time *t* = *t*_2_, with *t*_2_ > *t*_1_. They are given by:

$$\mu =\frac{1}{N}\mathop{\sum }\limits_{i=1}^{N}{\delta }_{{x}_{i}}\,{\rm{and}}\,\nu =\frac{1}{N}\mathop{\sum }\limits_{j=1}^{N}{\delta }_{{y}_{j}},$$

where *δ* is the Dirac distribution, ${x}_{i}={({P}_{g}^{i}({t}_{1}))}_{g=1,\ldots ,G}$ is the level of gene *g*’s protein in cell *x*_*i*_ at *t*_1_ and ${y}_{j}={({P}_{g}^{j}({t}_{2}))}_{g=1,\ldots ,G}$ is the level of gene *g*’s protein in cell *y*_*j*_ at *t*_2_. For the sake of simplicity, when using simulated data cells have the same index in distributions *μ* and *ν* (i.e., the *i*-th component of *μ* is associated with the same cell than the *i*-th component in *ν*). Additionally, the two distributions are jointly normalized (between 0 and 1). Default parameter values are given in Table 1.

### Reduced model with analytical solution

In addition to the model introduced in the previous section, for which couplings will be estimated from simulations, we consider a simplified model (reduced Bursty model) that will provide closed-form couplings. This simplification is achieved in two steps. First, a protein-only model is derived from a time scale separation of mRNA and proteins corresponding to *d*_0_ ≫ *d*_1_ (i.e., proteins assumed to be more stable than mRNAs). This model is defined by refs. ^49,50^:

3 $$\left\{\begin{array}{l}P(t)\mathop{\to }\limits^{{k}_{on}}P(t)+{\mathcal{E}}(1/\eta )\\ \dot{P}(t)=-{d}_{1}P(t)\end{array}\right.$$

where ${\mathcal{E}}$ is the exponential distribution characterizing the random size of each burst (with mean value *η*), *k*_on_ is the burst rate and *d*_1_ the degradation rate of proteins, as before. Second, we consider a particular 1-dimensional case of model (3) with a constant *k*_on_: this setting can be interpreted either as a single gene with no feedback, or part of a GRN within a time range sufficiently short so that *k*_on_(*P*_1_, …, *P*_*G*_) does not vary significantly during this period.

This model turns out to be analytically tractable (see Supplementary File, Section 1). The time-dependent distribution of *P*(*t*) knowing *P*(0) = *P*_0_ is given by

4 $${\mathcal{L}}(P(t)| P(0)={P}_{0})={e}^{-{k}_{on}t}{\delta }_{{e}^{-{d}_{1}t}{P}_{0}}+(1-{e}^{-{k}_{on}t}){\nu }_{t}$$

where ${\delta }_{{e}^{-{d}_{1}t}{P}_{0}}$ is the Dirac measure localized at ${e}^{-{d}_{1}t}{P}_{0}$, and *ν*_*t*_ has a density function *g*_*t*_(*x*∣*P*_0_) given by

5 $${g}_{t}(x| {P}_{0})={f}_{t}(x-{e}^{-{d}_{1}t}{P}_{0}),$$

where

$${f}_{t}(x)=\frac{1}{\eta }\frac{({e}^{{d}_{1}t}-1){k}_{on}}{({e}^{{k}_{on}t}-1){d}_{1}}\exp \left(-\frac{x}{\eta }{e}^{{d}_{1}t}\right)\Phi \left(\frac{{k}_{on}}{{d}_{1}}+1;2;({e}^{{d}_{1}t}-1)\frac{x}{\eta }\right){{\mathbb{1}}}_{\{x\ge 0\}},$$

and *Φ* denotes the confluent hypergeometric (Kummer’s) function of the first kind.

For numerical applications, we regularize the singular part in (4) by replacing it with a normal distribution with small standard deviation *ε* ≪ 1,

6 $${\mathcal{L}}(P(t)| P(0)={P}_{0})\approx {e}^{-{k}_{on}t}{\mathcal{N}}({e}^{-{d}_{1}t}{P}_{0},{\varepsilon }^{2})+(1-{e}^{-{k}_{on}t}){\nu }_{t},$$

so that the equality holds when *ε* → 0.

### Coupling between time points

Let us recall that experimental data would be collections of cell positions in the protein space at distinct time points, and that the objective of cell trajectory inference is to reconstruct cell trajectories in this space. Hence, we restrict the problem to two consecutive time points *t*_1_ and *t*_2_. Given two collections $X=({x}_{1},\ldots ,{x}_{N})\in {({{\mathbb{R}}}_{+}^{G})}^{N}$ and $Y=({y}_{1},\ldots ,{y}_{N})\in {({{\mathbb{R}}}_{+}^{G})}^{N}$, we search to construct a matrix $Q=({Q}_{ij})\in {{\mathbb{R}}}_{+}^{N\times N}$ of probabilities *Q*_*ij*_ that a cell at position *x*_*i*_ at time *t*_1_ reaches the state *y*_*j*_ at time *t*_2_, in the most realistic way. In the following, the matrix *Q* will be called a coupling between the collections *X* and *Y*.

By solving the Schrödinger problem (see Section Schrödinger problem), we look for the coupling that is the closest to a certain reference coupling $R=({R}_{ij})\in {{\mathbb{R}}}_{+}^{N\times N}$, obtained using the Bursty model (1)-(2), while being compatible with the data *X* and *Y*. For either the model (1)-(2) or the Diffusive model, the reference coupling *R* is built by associating with the model a function being an exact or an approximated version of the transition kernel of the underlying theoretical process.

#### **Coupling for the reduced Bursty model**

In the case of a single gene following model (3), we use the regularized analytical solution (6) to compute the probability density of reaching state *y* starting from *x*, that is *p*_*t*_(*y*∣*x*) defined by

$${p}_{t}(y| x)={e}^{-{k}_{on}t}{h}_{t}(y| x)+(1-{e}^{-{k}_{on}t}){g}_{t}(y| x)$$

where *g*_*t*_ is defined in (5) and

$${h}_{t}(y| x)=\frac{1}{\sqrt{2\pi {\varepsilon }^{2}}}\exp \left\{-\frac{{(y-{e}^{-{d}_{1}t}x)}^{2}}{2{\varepsilon }^{2}}\right\}.$$

Considering this transition kernel *p*_*t*_(*y*∣*x*) and the uniform distribution *p*(*x*) ≡ 1/*N* over initial states (*x*_1_, …, *x*_*N*_), the related reference coupling *RB*_ref_(*x*, *y*), for “reduced Bursty reference coupling”, is defined as:

$$R{B}_{{\rm{ref}}}(x,y)=\frac{1}{N}\frac{{p}_{{t}_{2}-{t}_{1}}(y| x)}{c(x)}$$

where the normalizing term $c(x)={\sum }_{1\le j\le N}{p}_{{t}_{2}-{t}_{1}}({y}_{j}| x)$ is defined so that ${p}_{{t}_{2}-{t}_{1}}(y| x)/c(x)$ is a discrete probability distribution over (*y*_1_, …, *y*_*N*_).

#### **Coupling for the non-reduced Bursty model**

Given a GRN whose dynamics are given by a Bursty model (1)-(2), we construct a corresponding approximated coupling *B*_ref_(*x*, *y*), denoted most of the time *B*_ref_, an approximation of its coupling between two collections at two different time points. It is the reference coupling defined by the probability of reaching *y* when starting from *x*, computed from the Bursty model (1)-(2).

**Fig. 13:**
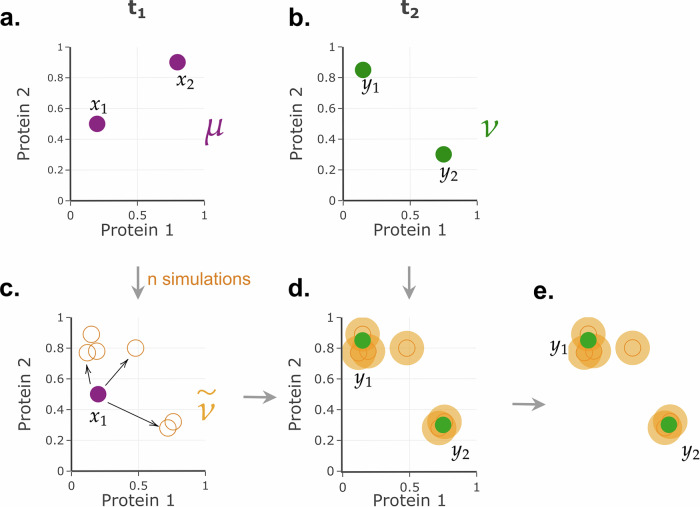
Schematic of the procedure used for constructing the *B*_ref_ estimation with a Gaussian method. **a** In this example, *N* = 2 and cells *x*_1_ and *x*_2_ (purple dots) are the two cells of the distribution *μ* at time *t*_1_ in protein space. **b** Cells *y*_1_ and *y*_2_ (green dots) are the two cells belonging to the distribution *ν* at time *t*_2_ in the protein space. **c** The approximated distribution $\widetilde{\nu }$ is computed for *n* = 6 and cells are displayed as orange circles. **d** Gaussian distributions centered on $\widetilde{\nu }$ cells generate a smooth distribution (orange areas). **e** Probabilities *B*_*ref*_(*y*_1_∣*x*_1_) and *B*_*ref*_(*y*_2_∣*x*_1_) are deduced from the construction process.

We estimate *B*_ref_ using a Gaussian method (illustrated in Fig. 13). For each cell *i* in the initial distribution *μ* at *t* = *t*_1_, we simulate the Bursty model (1)-(2)*n* times with the cell *i*’s coordinates as an initial condition with the same parameters than those used to compute the simulated data detailed in Table 1 (with one exception, when we test the influence of GRN parameter values in Section Unspecificity of the entropy indicator and the distribution parameters: in this case, the parameter values used to compute the couplings are the ones of the tested GRN, not the ones used to generate simulated data). We obtain a distribution denoted $\widetilde{\nu }$ at time *t* = *t*_2_, representing the potential fates of cell *i*. An estimated density of $\widetilde{\nu }$ is computed using a Gaussian approximation, i.e., we create Gaussian distributions centered on cells in the distribution $\widetilde{\nu }$, and we compute the Gaussian kernel of this distribution using the default parameters of the *gaussian_kde()* python function. The result is then divided by the total sum to ensure the resulting distribution is a probability distribution.

#### **Coupling for a diffusive process**

When the underlying model is a Diffusive process, we consider the natural discrete counterpart used in ref. ^6^. Specifically, the reference coupling denoted by *D*_ref_ is defined by

$${D}_{{\rm{ref}}}(x,y)=\frac{1}{{\bar{D}}_{{\rm{ref}}}}\exp \left(-\frac{\parallel x-y{\parallel }^{2}}{2{\sigma }^{2}({t}_{2}-{t}_{1})}\right)$$

where *σ*^2^ is the diffusion coefficient, ∥*x* − *y*∥ is the Euclidean distance (here, in protein space) between *x* and *y*, and ${\bar{D}}_{{\rm{ref}}}$ is the normalizing constant given by

$${\bar{D}}_{{\rm{ref}}}=\mathop{\sum }\limits_{1\le i,j\le N}\exp \left(-\frac{\parallel {x}_{i}-{y}_{j}{\parallel }^{2}}{2{\sigma }^{2}({t}_{2}-{t}_{1})}\right).$$

The value of the diffusion coefficient was chosen to be both relevant and close to the value used in ref. ^5^. The challenge was to consider a *σ* large enough to ensure a fast convergence of the Sinkhorn algorithm, while still capturing sufficiently accurate couplings (*D*_ref_(*x*, *y*) > 0).

### Schrödinger problem

In its modern formulation, the Schrödinger problem, first introduced in 1932^51^, aims at minimizing the relative entropy of a coupling with respect to a reference coupling, with marginal constraints. This problem is deeply linked with OT: when the reference coupling is the joint law of a reversible Brownian motion with a given diffusion coefficient between times *t*_1_ and *t*_2_, then the Schrödinger problem converges towards quadratic OT as the diffusion coefficient goes to 0^52,53^.

Let *Π* and *Ψ* be two discrete and finite spaces, e.g. *Π* = {*x*_1_, …, *x*_*N*_} and *Ψ* = {*y*_1_, …, *y*_*N*_} with $N\in {\mathbb{N}}$, the number of cells. We denote by $R\in {\mathcal{P}}(\Pi \times \Psi )$, the reference process, where ${\mathcal{P}}(\Pi \times \Psi )$ is the space of probability measures on *Π* × *Ψ*.

Since *Π* and *Ψ* are discrete spaces of size *N*, then *R* is a *N* × *N* matrix. We denote by $u\in {\mathcal{P}}(\Pi )$ and $v\in {\mathcal{P}}(\Psi )$ two uniform vectors of size *N* (each cell has the same weight), and we search for the process *Q* solution of

7 $$Sch(R;u,v)=\mathop{\arg \,\min }\limits_{Q\in {\mathcal{P}}(\Pi \times \Psi )}\left\{H(Q| R);\,\forall i,\,{u}_{i}=\mathop{\sum }\limits_{j}{Q}_{ij},\,\forall j,\,{v}_{j}=\mathop{\sum }\limits_{i}{Q}_{ij}\right\},$$

where *H* is the relative entropy of *Q* with respect to *R* (also called Kullback–Leibler divergence of *Q* from *R*) defined by

8 $$H(Q| R)=\mathop{\sum }\limits_{ij}{Q}_{ij}\log \left(\frac{{Q}_{ij}}{{R}_{ij}}\right).$$

This quantity will be called entropy throughout this paper. The Schrödinger problem (7) can be solved with the Sinkhorn algorithm^18,19^.

We solve the Schrödinger problem for the reference couplings introduced in Section Coupling between time points. When *R* = *RB*_ref_, we denote by *RB*_sch_ its solution, *RB*_sch_ ≔ *Sch*(*RB*_ref_; *u*, *v*); for the reference coupling *R* = *B*_ref_, we denote by *B*_sch_ its solution, *B*_sch_ ≔ *Sch*(*B*_ref_; *u*, *v*); and when *R* = *D*_ref_, then we denote *D*_sch_ its solution, *D*_sch_ = *Sch*(*D*_ref_; *u*, *v*).

### Performances of the models

In order to compare the performances of the Bursty model-based inference method with the Diffusion-based inference method, we rely on the computation of a standard criterion, the entropy (8) between solutions of the Schrödinger problem (7). However, to ensure that results do not depend on the criterion, we also test other criteria measuring the difference between two stochastic processes.

**Entropy between Schrödinger problem solutions**. For one-gene GRN, we define

$${H}_{B,R}:=H({B}_{{\rm{sch}}}| R{B}_{{\rm{sch}}})\,{\rm{and}}\,{H}_{D,R}:=H({D}_{{\rm{sch}}}| R{B}_{{\rm{sch}}}),$$

i.e., the entropy between *B*_sch_ or *D*_sch_ and *RB*_sch_. These values characterize the ability of a Diffusive process or a Bursty model versus *RB*_sch_ to predict cell trajectories correctly. The process *RB*_sch_ is considered the reference process because it provides an approximation of the exact probability distribution. In parallel, we compute as a control measure

$${H}_{R}:=H(R{B}_{{\rm{ref}}}| R{B}_{{\rm{sch}}})$$

in order to estimate the error made when performing stochastic simulations.

For two-or-more-gene GRN, we define

$${H}_{D,B}:=H({D}_{{\rm{sch}}}| {B}_{{\rm{sch}}}),$$

the entropy between *D*_sch_ and *B*_sch_, in order to evaluate the difference between the ability of a Diffusive process versus a Bursty model to correctly predict cell trajectories. Similarly to the one-gene GRN case above, we introduce

$${H}_{B}:=H({B}_{{\rm{ref}}}| {B}_{{\rm{sch}}})$$

to estimate the error made when performing stochastic simulations.

Values of *H*_*B*_ should be smaller than *H*_*D*,*B*_ values if *B*_ref_ and *B*_sch_ are closer than *D*_sch_ and *B*_sch_. If the Bursty model-based reference process better predicts individual cell trajectories, the difference between *H*_*B*_ and *H*_*D*,*B*_ will be important.

Due to stochasticity, we perform 100 iterations to compute these quantities and focus on the mean and standard deviation.

**Other difference indicators**. To certify that our results do not depend on the choice of the entropy, we also compute the following criteria, for two processes *Q* and *R*:

- the Total Variation Distance:
  $${D}_{{\rm{TV}}}(Q,R)=\frac{1}{2}\mathop{\sum }\limits_{i}\mathop{\sum }\limits_{j}| {Q}_{ij}-{R}_{ij}| ;$$
- the Jensen–Shannon Divergence:
  $${\rm{JSD}}(Q,R)=(H(Q\,| \,W)+H(R\,| \,W))/2,$$
  where *W* = (*Q* + *R*)/2;
- the Frobenius norm:
  $$\parallel Q-R{\parallel }_{F}=\sqrt{\mathop{\sum }\limits_{i,j}{({Q}_{ij}-{R}_{ij})}^{2}}.$$

### Application to ref. ^38^ dataset

We use a time-stamped in vitro dataset from ref. ^38^ made of mRNA counts obtained from scRNA-seq experiments on mouse embryonic stem cells. The dataset comprises 41 genes whose levels of expression were measured at 9 observation times (0, 6, 12, 24, 36, 48, 60, 72, and 96 h) with approximately hundreds of cells obtained at each observation time. Genes are listed in Fig. 9. Ventre et al.^36^ inferred a GRN based on the Bursty model driving the dynamics observed in the dataset. We use this GRN and the associated parameter values to compute the reference coupling *B*_ref_, similarly to what has been done with protein inputs, but using mRNA counts. Compared to the work done on simulated protein data, we use a larger number of simulations when constructing the approximated reference process, *n* = 10,000, while other parameter values are given in Table 1.

Given two experimental distributions *μ* at time *t*_1_ and *ν* at time *t*_3_, we hereafter describe how we predict the distribution at time *t*_2_ ∈ (*t*_1_, *t*_3_).

By simulating the Bursty model with the GRN from ref. ^36^, we first compute *B*_ref_(*t*_1_, *t*_3_), the reference coupling between times *t*_1_ and *t*_3_, and a reference distribution *ρ*_ref_ at time *t*_2_. Second, we solve the Schrödinger problem and determine the coupling *B*_sch_(*t*_1_, *t*_3_). We sample cell pairs (*x*, *y*) based on the coupling *B*_sch_(*t*_1_, *t*_3_): the goal is to generate a certain number of pairs for each cell *x* in *μ*, where the probability of selecting a cell *y* in *ν* is determined by *B*_sch_(*t*_1_, *t*_3_). Third, we build an interpolated distribution at *t*_2_, rooted in distributions *μ* and *ν*, as described below.

We define the estimated, interpolated distribution at time *t*_2_ as

9 $${\rho }_{{\rm{est}}}:={T}_{{t}_{2}}^{{\alpha }^{* }}\#{B}_{{\rm{sch}}}({t}_{1},{t}_{3}).$$

with ${T}_{{t}_{2}}^{{\alpha }^{* }}:(x,y)\mapsto {\alpha }^{* }x+(1-{\alpha }^{* })y$ for each pair (*x*, *y*), ${\alpha }^{* }\in {{\mathbb{R}}}^{G}$ is an optimized vector and *#* is the push-forward function. Details of the optimization procedure leading to the estimation of α^*^ are provided in the Supplementary File. We compare the estimated distribution *ρ*_est_ with the experimental distribution, using the Earth mover’s distance (EMD), denoted by EMD_*B*_. We also compute the coupling between the distributions *μ* at *t*_1_ and *ν* at *t*_3_ using regularized OT (similarly to WOT^5^), denoted by *D*_sch_(*t*_1_, *t*_3_). In this case, we use a log-transform of mRNA counts, we do not account for growth and death since this is not relevant in this case, and we interpolate a distribution at *t*_2_ as in ref. ^5^ by using α^*^ = (*t*_2_ − *t*_1_)/(*t*_3_ − *t*_1_) = 0.5 in (9) for all genes. We then compare the interpolated distribution and the experimental distribution by computing the EMD, denoted by EMD_*D*_. Finally, we compare the two distances, EMD_*B*_ and EMD_*D*_, by computing

$${\Delta }_{EMD}=EM{D}_{D}-EM{D}_{B}.$$

## Supplementary information

Supplementary information

## Data Availability

All results included in this manuscript can be reproduced using the codes mentioned below. Experimental data used in this work have been provided by Semrau et al. (2017) and are accessible at https://www.ncbi.nlm.nih.gov/geo/ under accession number GSE79578.

## Code availability

All Python codes are available at https://github.com/fourniecl/inference_trajectories_PDMP.
