# Supplementary material for: Cell trajectory inference based on schrödinger problem and a mechanistic model of stochastic gene expression
Source: NPJ Syst Biol Appl. 2026 May 21;12:120. doi: 10.1038/s41540-026-00743-x (PMC13463083; doi:10.1038/s41540-026-00743-x)
Supplement: Supplementary file 1 [file 41540_2026_743_MOESM1_ESM.pdf]

Cell Trajectory Inference based on Schrödinger problem and a mechanistic model of stochastic gene expression

Supplementary File

Clémence Fournié, Elias Ventre, Ulysse Herbach, Aymeric Baradat, Olivier Gandrillon, Fabien Crauste

1 Analytical solution for the reduced Bursty model

Let us consider the reduced Bursty model

$$\begin{cases} P(t) \xrightarrow{k_{on}} P(t) + \mathcal{E}(1/\eta), \\ \dot{P}(t) = -d_1 P(t), \end{cases}$$

where $P(t)$ is the protein level at time t, $\mathcal{E}$ is the exponential distribution characterizing the random size of each burst (with mean value η), k_{on} is the burst rate, assumed to be constant, and d_1 is the degradation rate of proteins.

We briefly explain how to derive the formula

$$\mathcal{L}(P(t)|P(0) = P_0) = e^{-k_{on}t}\delta_{e^{-d_1 t}P_0} + (1 - e^{-k_{on}t})\nu_t$$

where $\delta_{e^{-d_1 t}P_0}$ is the Dirac measure localized at $e^{-d_1 t}P_0$, and $\nu_t = \delta_{e^{-d_1 t}P_0} * \mu_t$ with μ_t the probability measure defined by the following density function:

$$f_t(x) = \frac{1}{\eta}\frac{(e^{d_1 t} - 1)k_{on}}{(e^{k_{on}t} - 1)d_1} \exp\left(-\frac{x}{\eta}e^{d_1 t}\right) \Phi\left(\frac{k_{on}}{d_1} + 1; 2; (e^{d_1 t} - 1)\frac{x}{\eta}\right) \mathbb{1}_{\{x \geq 0\}}, \tag{1}$$

where Φ denotes the confluent hypergeometric (Kummer's) function of the first kind. Hence the probability measure ν_t has a density function $g_t(x|P_0)$ given by

$$g_t(x|P_0) = f_t(x - e^{-d_1 t}P_0). \tag{2}$$

The corresponding Markov process $(X_t)_{t\geq 0}$, with values in $\mathbb{R}_+$, is characterized by its infinitesimal generator L, defined in Malrieu (2015) *Some simple but challenging Markov processes,* Annales de la Faculté de Sciences de Toulouse, 24, 857–883,

$$Lf(x) = -d_1 x f'(x) + k_{on} \int_0^\infty (f(x+h) - f(x)) \frac{1}{\eta} e^{-\frac{1}{\eta}h} \mathrm{d}h,$$

from which an evolution equation for the Laplace transform $\phi(s,t) = \mathbb{E}[e^{-sX_t}]$ can be derived, leading to the general solution

$$\phi(s,t) = \phi(se^{-d_1 t}, 0) \left(\frac{1 + \eta e^{-d_1 t} s}{1 + \eta s} \right)^{\frac{k_{on}}{d_1}}.$$

The first term in this product can be identified as the Laplace transform of $e^{-d_1 t} X_0$, while the second term is the Laplace transform of X_t conditional on $X_0 = 0$ (i.e., $\mathcal{L}(X_t | X_0 = 0)$). It turns out that the latter can be inverted explicitly using a classical series known as the confluent hypergeometric function of the first kind (denoted by $\Phi(a; b; z)$), which is of practical interest since this special function is implemented in standard computing libraries such as SciPy. Manipulation of the series leads to the probability measure

$$\mathcal{L}(X_t | X_0 = 0) = e^{-k_{on} t} \delta_0 + (1 - e^{-k_{on} t}) \mu_t,$$

where μ_t has density function $f_t(x)$ given in (1), and the final expression (1)-(2) follows.

Interestingly, the weight of the singular part (Dirac measure at $e^{-d_1 t} P_0$) decreases exponentially with rate k_{on}, in line with the property that in this model, a trajectory ceases to be deterministic precisely when the first burst occurs.

2 Sensitivity results

Supp. Figure 1 shows sensitivity results for some parameters of the Bursty model-based inference method. We focused on the impact on the Entropy $H_{D,B}$ of the number of simulations used to compute B_{ref} (Supp. Figure 1a.), the standard deviation ε of the Gaussian law in (6) (Supp. Figure 1b.), the precision of Sinkhorn algorithm (Supp. Figure 1c.) and the truncation used to avoid zeros in Sinkhorn algorithm (Supp. Figure 1d.). Results highlight a weak sensitivity to variations in these parameters, as long as their values remain reasonable.

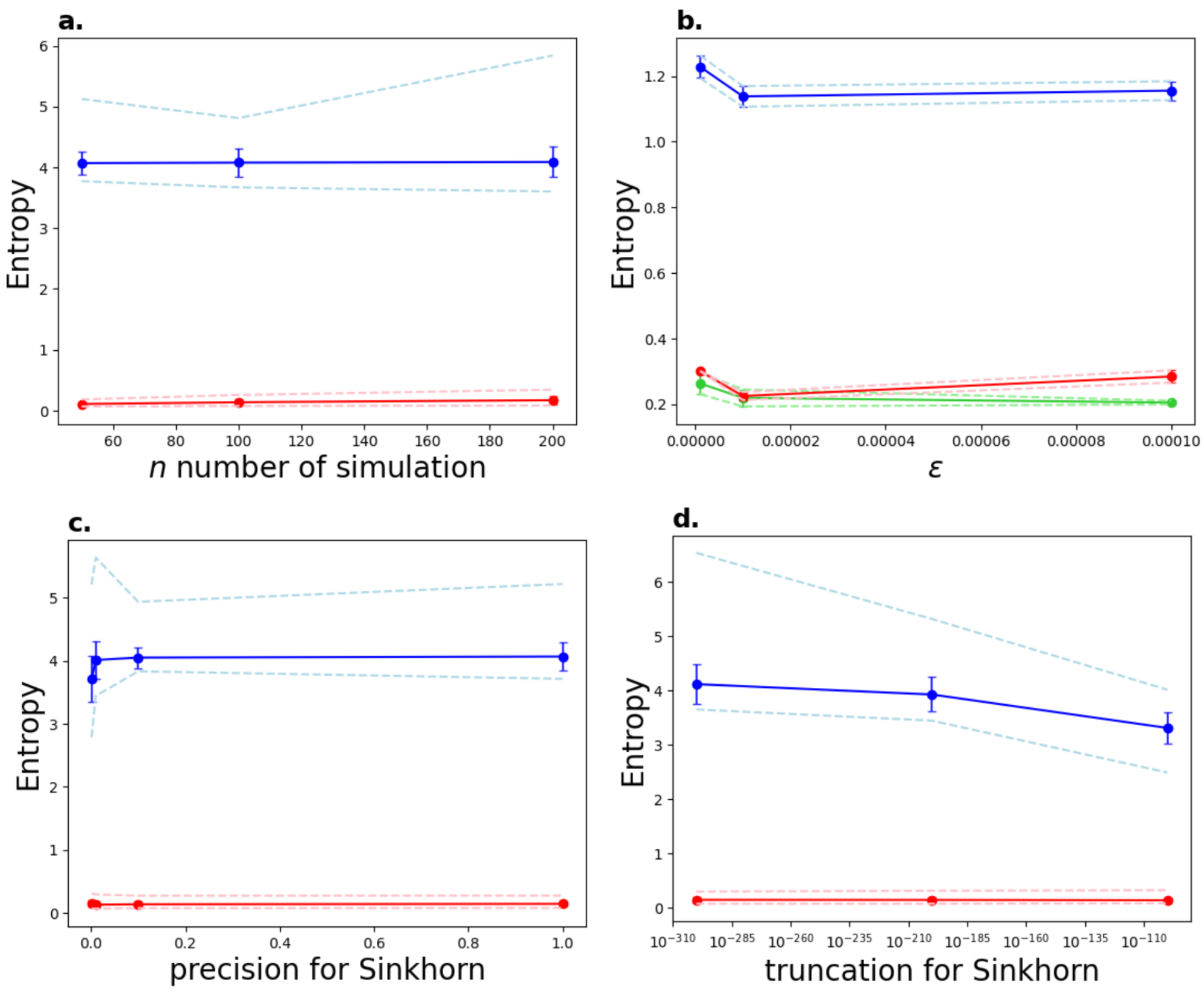

Supp. Figure 1: **Entropy as a function of hyperparameters.** Mean (dots), standard deviation, min and max values (dashed lines) of $H_{D,B}$ (blue), H_B (red) and H_R (green, only in **b.**) are represented as functions of **a.** the number of simulations used to compute B_{ref}; **b.** ε, the standard deviation of the Gaussian law in (6); **c.** the precision of Sinkhorn algorithm; **d.** the truncation used to avoid zeros in Sinkhorn algorithm. All other parameters are fixed and given in Table 1 (main text).

Supp. Figure 2 shows sensitivity results for GRN parameters β_i the basal activity rate, the half-life of mRNA $\log(2)/d_{0,i}$, and the half-life of proteins $\log(2)/d_{1,i}$, of gene i (note that all genes in the Toggle Switch are assumed to have the same parameter values). Their impact on the entropies $H_{D,B}$ and H_B is limited.

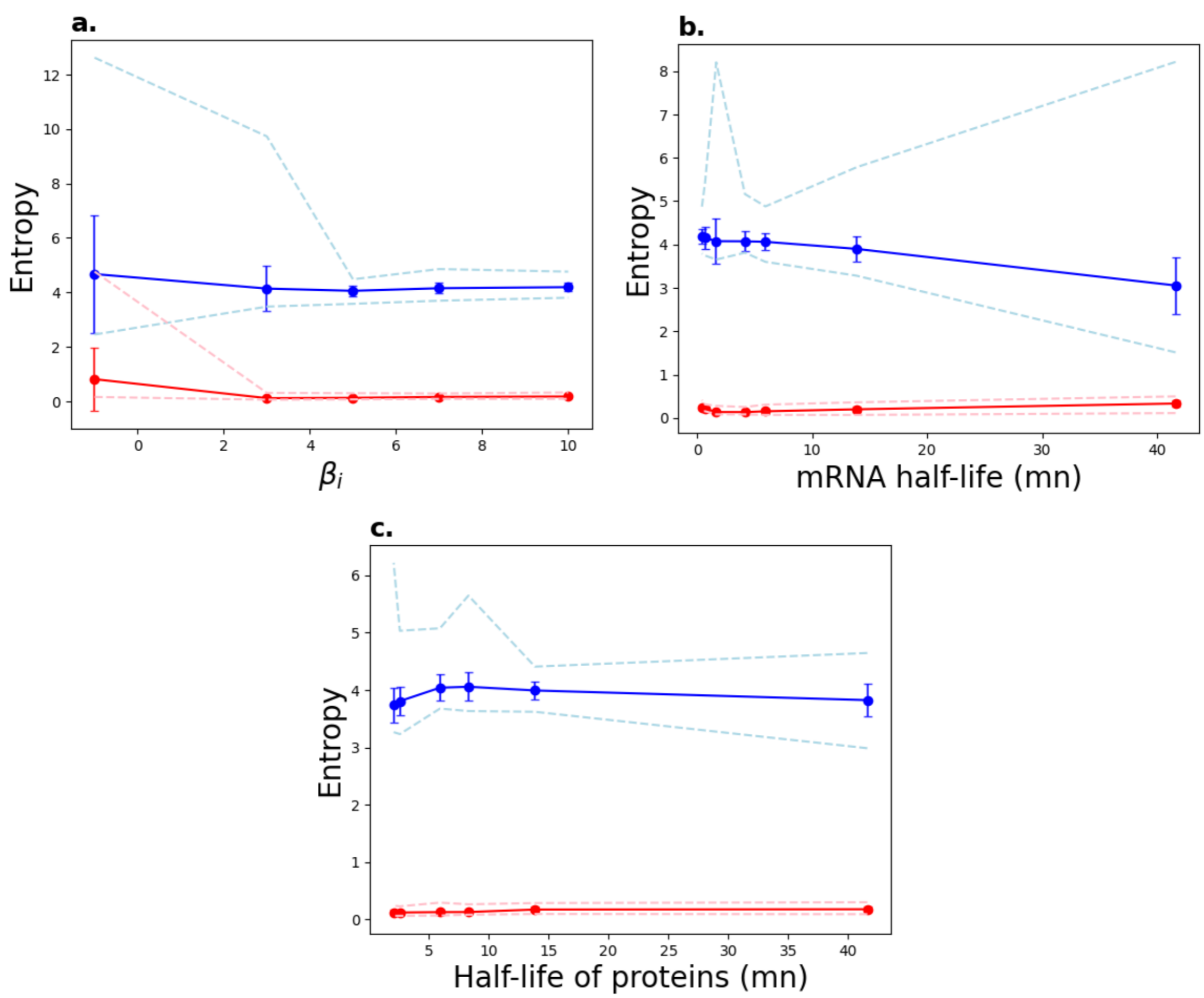

Supp. Figure 2: **Entropy as a function of GRN parameters**. Mean (dots), standard deviation, minimum and maximum values (dashed lines) of $H_{D,B}$ (blue) and H_B (red) as functions of **a.** the rate of basal activity, β_i; **b.** the half-life of mRNA, $\log(2)/d_{0,i}$; **c.** the half-life of proteins, $\log(2)/d_{1,i}$, of gene i (all genes have the same parameter values by default). All other parameters are fixed and given in Table 1 (main text).

3 The stationary case

We studied the influence of the time difference $t_2 - t_1$ on the solutions of the Schrödinger problem when the system is in a stationary state. To do so, we defined $t_{1,st} = 40$ h and $t_{2,st} \in [42; 64]$ h. Results are displayed in Supp. Figure 3.

Results are similar to the transient state case, except that the $H_{D,B}$ curve decreases as $t_{2,st}$ increases. This shows that despite the Diffusive model being less accurate than the Bursty model to infer trajectories, their performances become comparable when the final distribution becomes independent of the initial one, that is for large $t_{2,st}$ values.

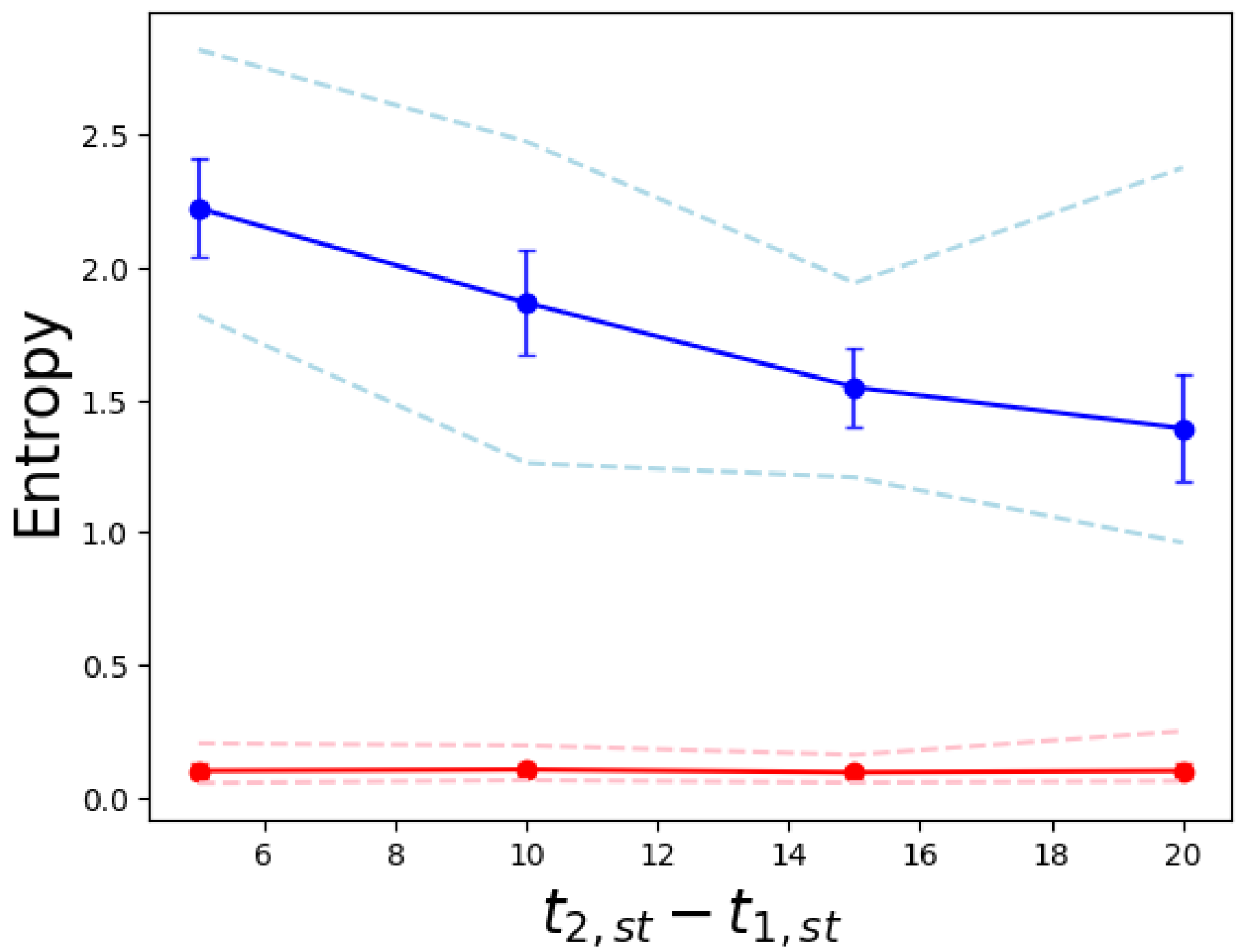

Supp. Figure 3: **Entropies between reference couplings for the toggle switch GRN, in the stationary state.** Mean and standard deviation (straight lines) of $H_{D,B}$ (blue) and H_B (red) are displayed as functions of the time interval $t_{2,st} - t_{1,st}$. Additionally, minimum and maximum values over 100 iterations are represented (dashed lines). Other parameters are given in Table 1 (main text).

4 Interpolation parameter α and empirical measures

The interpolation application at time t_2 is defined as

$$T_{t_2}^{\alpha} : (x, y) \mapsto \alpha x + (1-\alpha) y, \quad \alpha \in \mathbb{R}^G. \tag{3}$$

The optimized value of α, denoted by α^*, is obtained as the solution of a minimisation problem:

$$\alpha^* = \arg\min_{\alpha} \ \mathcal{L}(\alpha),$$

where the cost function $\mathcal{L}$ is the quadratic Wasserstein distance between the empirical distribution ρ_{ref} at t_2 and the empirical interpolated distribution $T_{t_2}^{\alpha} \# B_{\text{ref}}(t_1, t_3)$, given by

$$\mathcal{L}(\alpha) := W_2^2 \left(T_{t_2}^{\alpha} \# B_{\text{ref}}(t_1, t_3), \rho_{\text{ref}} \right).$$

The optimization is performed with the Python package geomloss, using the SamplesLoss function (`https://www.kernel-operations.io/geomloss/index.html`), and minimization is done with PyTorch.

Once α^* is found, the estimated distribution at time t_2 is defined as

$$\rho_{\text{est}} := T_{t_2}^{\alpha^*} \# B_{\text{sch}}(t_1, t_3).$$

In practice, we consider empirical measures. The push-forward function of the reference coupling by $T_{t_2}^{\alpha}$ is given explicitly by

$$T_{t_2}^{\alpha} \# B_{\text{ref}}(t_1, t_3) = \frac{1}{N} \sum_{i=1}^{N} \delta_{\alpha x_i + (1-\alpha) y_i},$$

where N is the number of sampled pairs (x_i, y_i) from the coupling $B_{\text{ref}}(t_1, t_3)$, that is N equals the number of cells x times the number of samples for each cell. δ denotes the Dirac measure.

Similarly, the reference distribution at time t_2 writes

$$\rho_{\text{ref}} = \frac{1}{m} \sum_{j=1}^{m} \delta_{z_j}, \qquad z_j \in \mathbb{R}^G,$$

with m depending on the number of simulated vectors from the initial distribution μ.

5 Comparison of the Bursty model-based inference method and the Diffusion-based inference method for the experimental data

Supp. Figure 4 shows the values of the Erath Mover's Distances (EMD) between the experimental data and the inference methods (Bursty model-based inference method in blue, Diffusion-based inference method in red). They are the EMD used to compute Δ_{EMD} in Figure 13.

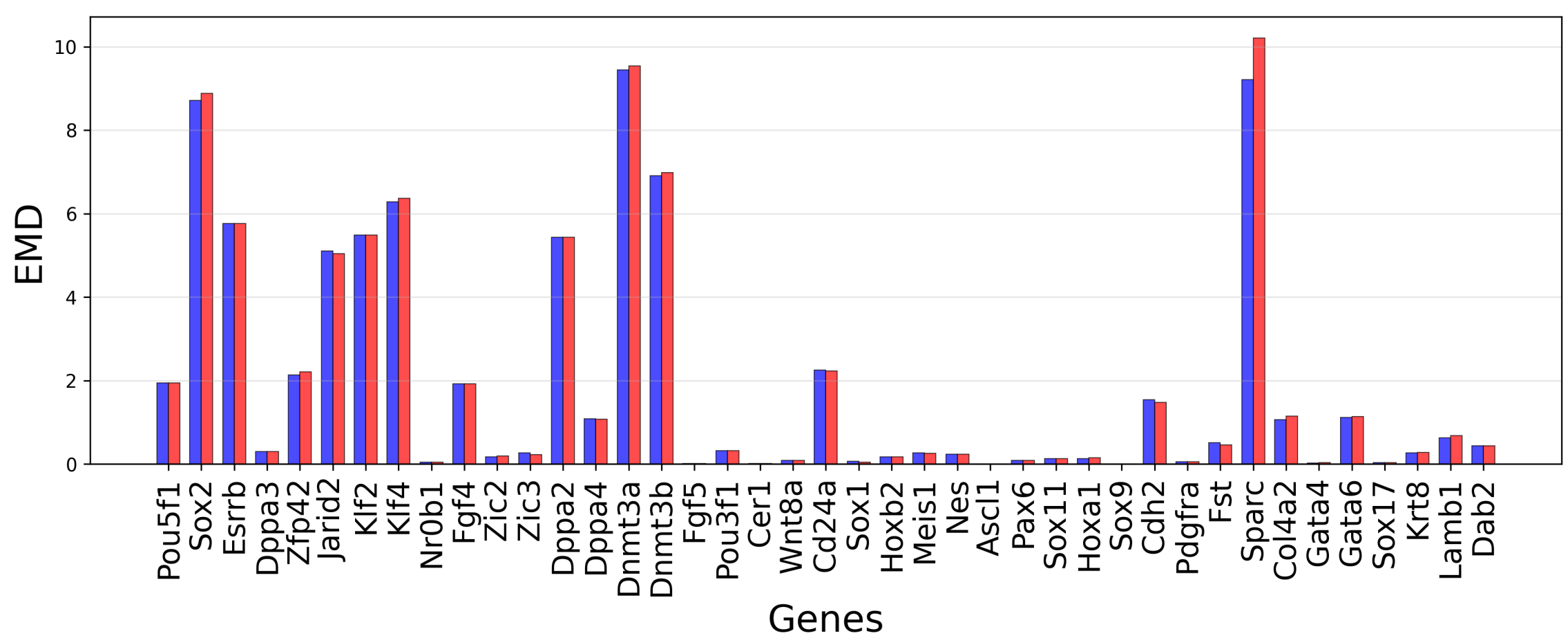

Supp. Figure 4: **EMD between Bursty model-based and Diffusion-based inference methods and experimental data for each gene in the dataset**. The EMDs are computed as described in Section 2.6, at the interpolated time $t_2 = 36$h, based on the coupling between times $t_1 = 0$h and $t_3 = 72$h. In blue are shown EMD between the Bursty model-based prediction and the experimental data, and in red the EMD between the Diffusion-based prediction and the experimental data.
